# G protein-coupled estrogen receptor stimulates human trophoblast cell invasion via YAP-mediated ANGPTL4 expression

**DOI:** 10.1038/s42003-021-02816-5

**Published:** 2021-11-12

**Authors:** Jung-Chien Cheng, Lanlan Fang, Yuxi Li, Avinash Thakur, Pamela A. Hoodless, Yanjie Guo, Zhen Wang, Ze Wu, Yang Yan, Qiongqiong Jia, Yibo Gao, Xiaoyu Han, Yiping Yu, Ying-Pu Sun

**Affiliations:** 1 Center for Reproductive Medicine, Henan Key Laboratory of Reproduction and Genetics, The First Affiliated Hospital of Zhengzhou University, 450052 Zhengzhou, China; 2 Terry Fox Laboratory, BC Cancer Agency, Vancouver, BC Canada V5Z 1L3; 3 Department of Medical Genetics, University of British Columbia, Vancouver, BC Canada V6T 1Z4; 4 School of Biomedical Engineering, University of British Columbia, Vancouver, BC Canada V6T 1Z4

**Keywords:** Reproductive disorders, Reproductive disorders, Hormone receptors

## Abstract

Insufficient invasion of trophoblast cells into the uterine decidua is associated with preeclampsia (PE). G protein-coupled estrogen receptor (GPER) is a membrane estrogen receptor involved in non-genomic estrogen signaling. GPER is expressed in human trophoblast cells and downregulated GPER levels are noted in PE. However, to date, the role of GPER in trophoblast cells remains largely unknown. Here, we applied RNA sequencing (RNA-seq) to HTR-8/SVneo human trophoblast cells in response to G1, an agonist of GPER, and identified angiopoietin-like 4 (ANGPTL4) as a target gene of GPER. Treatment of trophoblast cells with G1 or 17β-estradiol (E2) activated Yes-associated protein (YAP), the major downstream effector of the Hippo pathway, via GPER but in a mammalian STE20-like protein kinase 1 (MST1)-independent manner. Using pharmacological inhibitors as well as loss- and gain-of-function approaches, our results revealed that YAP activation was required for GPER-stimulated ANGPTL4 expression. Transwell invasion assays demonstrated that activation of GPER-induced ANGPTL4 promoted cell invasion. In addition, the expression levels of GPER, YAP, and ANGPTL4 were downregulated in the placenta of patients with PE. Our findings reveal a mechanism by which GPER exerts its stimulatory effect on human trophoblast cell invasion by upregulating YAP-mediated ANGPTL4 expression.

## Abstract (web summary)

Cheng, Fan, Li et al. identified ANGPTL4 as a G1-induced target gene of GPER/YAP in HRT8 cells using RNA-seq and highlighted its importance in regulating trophoblast cell invasion. The authors also reported GPER downregulation in the placenta and lower estradiol levels in patients who developed preeclampsia.

## Introduction

17β-estradiol (E2), one of the main steroid hormones secreted by the ovaries, plays an essential role in the regulation of various reproductive functions. A significant increase in serum E2 levels can be observed during all trimesters of pregnancy, and aberrant regulation of E2 synthesis during pregnancy is associated with many pregnancy disorders^1,2^. E2 exerts its biological functions through genomic and non-genomic signaling pathways. The genomic signaling of E2 is mainly mediated by two nuclear estrogen receptors (ER), ERα and ERβ^3^. Non-genomic estrogen signaling is mediated by the G protein-coupled estrogen receptor (GPER), which is also known as GPR30^4^. Although serum levels of estrone (E1) and estriol (E3), particularly E3, are also increased throughout pregnancy, the affinity of E1 or E3 binding to GPER is much lower than that of E2^5^. Upon activation, GPER initiates many rapid responses, such as stimulating intracellular signaling pathways, classical second messengers, and calcium mobilization^6^. In humans, GPER is expressed in many types of tissue and is not restricted to E2-responsive tissues. Dysregulations of GPER expression and function are implicated in a diverse array of disorders, and targeting GPER has been considered a therapeutic approach for the treatment of several diseases^6,7^.

The human placenta is a highly invasive organ. During implantation, the placental trophoblast cells that are derived from the trophectoderm of the blastocyst start to proliferate, differentiate, and invade the underlying endometrial stroma to form chorionic villi. The chorionic villi are composed of two cell layers that are the outer syncytiotrophoblast (STB) layer and the inner cytotrophoblast (CTB) layer^8^. STB cells provide the interface between mother and fetus for nutrient transport and gas exchange in floating villi. Importantly, STB cells secrete critical hormones such as human chorionic gonadotropin and placental lactogen that are essential for normal pregnancy^9^. At the tips of the villi, CTB cells proliferation forms cell columns. The highly invasive extravillous cytotrophoblast (EVT) cells extended from cell columns invade the underlying maternal tissue and vasculature, thereby ensuring a continuous blood supply to the developing fetus throughout pregnancy^10^.

Placental disorders account for a number of poor reproductive outcomes. Aberrant placental development has severe consequences for the health of both the mother and fetus. In particular, insufficient trophoblast invasion and inadequate remodeling of the uterine vasculature are associated with the development of preeclampsia (PE) and fetal intrauterine growth restriction^11,12^. PE is a leading cause of maternal and perinatal mortality and morbidity that complicates 2–8% of pregnancies^13^. The most common features of PE are high blood pressure together with proteinuria that occurs after 20 weeks of gestation. During pregnancy, E2 is initially produced by the corpus luteum of ovaries for the first 9 weeks of gestation before the luteal–placental shift, where the primary source of production shifts to the placenta^2^. E2 can promote placental angiogenesis and uterine artery vasodilation. Many studies have reported that serum E2 levels are lower in women with PE compared with healthy pregnant women. In addition, the activities and expression levels of enzymes related to E2 synthesis are downregulated in placental cells of PE. Thus, insufficient E2 synthesis and/or signaling could lead to the development of PE and contribute to the pathogenesis of PE^14^.

GPER expression has been detected in various reproductive organs, including the placenta^4,15^. Interestingly, only a handful of studies have demonstrated that GPER expression levels are lower in the placenta of PE compared with that of gestational age-matched control placenta^16,17^. However, the physiological role of GPER in human trophoblast cells remains largely unknown, and how aberrant GPER expression affects the pathogenesis of PE needs to be explored. In this study, we applied transcriptome analysis to investigate the functional role of GPER in human trophoblast cells.

## Results

### RNA-seq identifies angiopoietin-like 4 (ANGPTL4) as a GPER target gene in human trophoblast cells

**Fig. 1:**
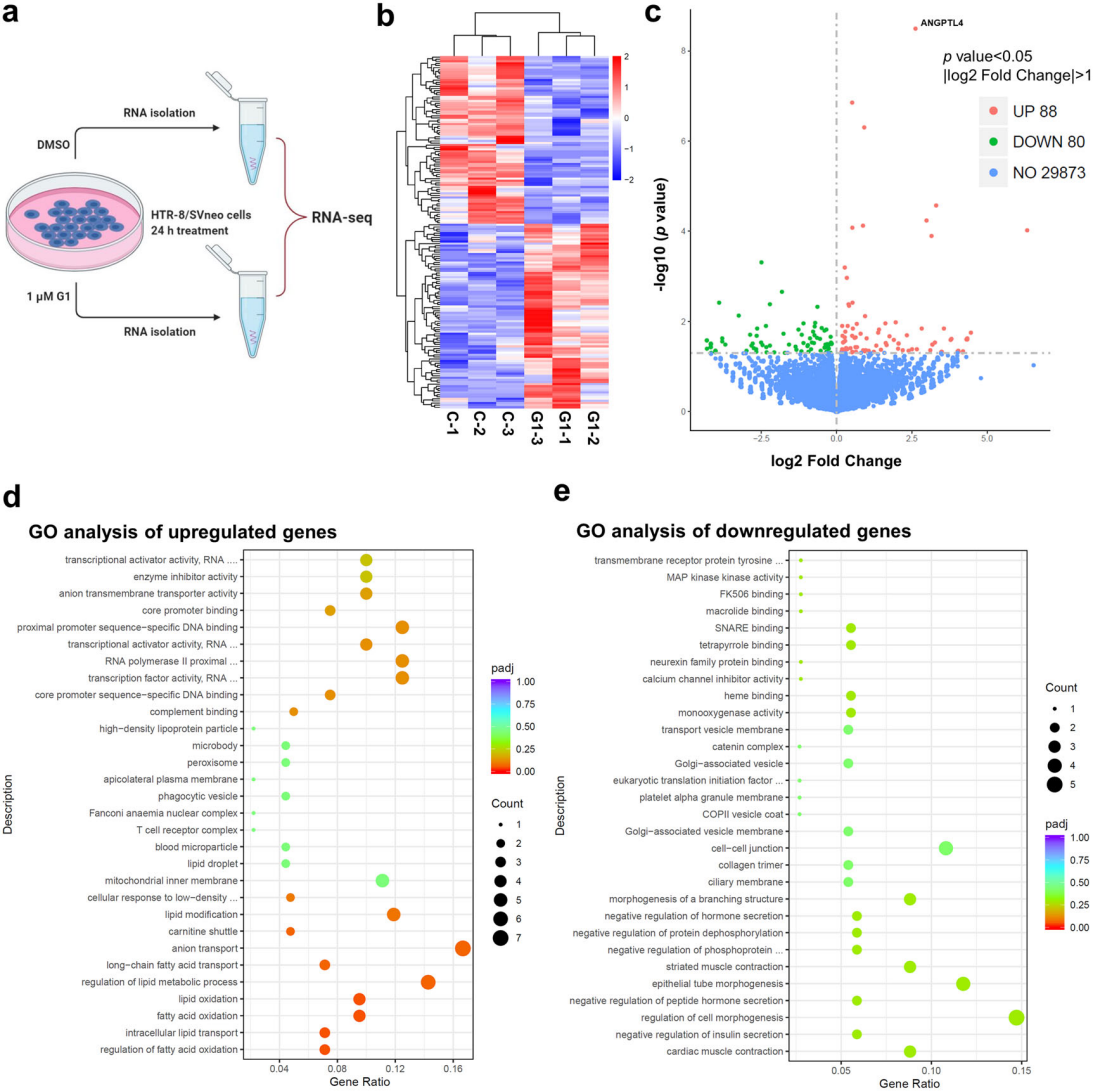
Transcriptome analysis of the effect of G1 on HTR-8/SVneo cells. **a** Schematic representation of RNA-seq experiment. **b** Cluster analysis of all the differentially expressed genes (DEGs) obtained from RNA-seq. **c** Volcano plot of RNA-seq data from vehicle control (DMSO)- and G1-treated cells. **d**, **e** A cluster profiler identified the enriched Gene Ontology (GO) processes of DEGs.

To explore the role of GPER in human trophoblast cells, a highly selective GPER agonist, G1, was used. G1 binds GPER with high affinity without activity toward ERα and ERβ^18^. RNA sequencing (RNA-seq) was performed on a human trophoblast cell line, HTR-8/SVneo, in triplicate after 24 h of G1 treatment (Fig. 1a). Transcriptome analysis revealed that the transcriptional levels of 168 genes were significantly affected by G1 treatment, of which 88 were upregulated and 80 were downregulated (Fig. 1b, c). The top 30 upregulated and downregulated genes by G1 treatment are presented in Supplementary Fig. 1. Gene Ontology (GO) analysis indicated that upregulated genes were enriched in the functions of anion transport, lipid metabolism, and lipid modification, whereas the downregulated genes were enriched in the regulation of cell morphogenesis and cell–cell junctions (Fig. 1d, e). Kyoto Encyclopedia of Genes and Genomes (KEGG) pathway analysis suggested that the upregulated genes were enriched in cholesterol metabolism and the peroxisome proliferator-activated receptor (PPAR) signaling pathway, whereas the downregulated genes were involved in pathways in retinol metabolism and endocytosis (Supplementary Fig. 2). These results suggest that GPER may play important roles in the regulation of placental nutrition and metabolism. Using RNA-seq data, we identified angiopoietin-like 4 (ANGPTL4) as a target gene of GPER in human trophoblast cells given that 6.14-fold upregulation of its mRNA level was observed after G1 treatment (Fig. 1c and Supplementary Fig. 1). ANGPTL4 was originally discovered in 2000 as a PPAR target gene. It belongs to a family of eight ANGPTL proteins that are structurally similar to angiopoietins and play important regulatory roles in a wide array of biological functions^19^. Pathologically, ANGPTL4 has been implicated in many disorders, such as metabolic and cardiovascular diseases, inflammation, and cancers^20,21^. Our RNA-seq results also showed that, except for ANGPTL5, which was not detected in HTR-8/SVneo cells, G1 treatment did not affect the mRNA levels of other ANGPTLs (Supplementary Fig. 3).

### Activation of GPER stimulates ANGPTL4 expression in human trophoblast cells

**Fig. 2:**
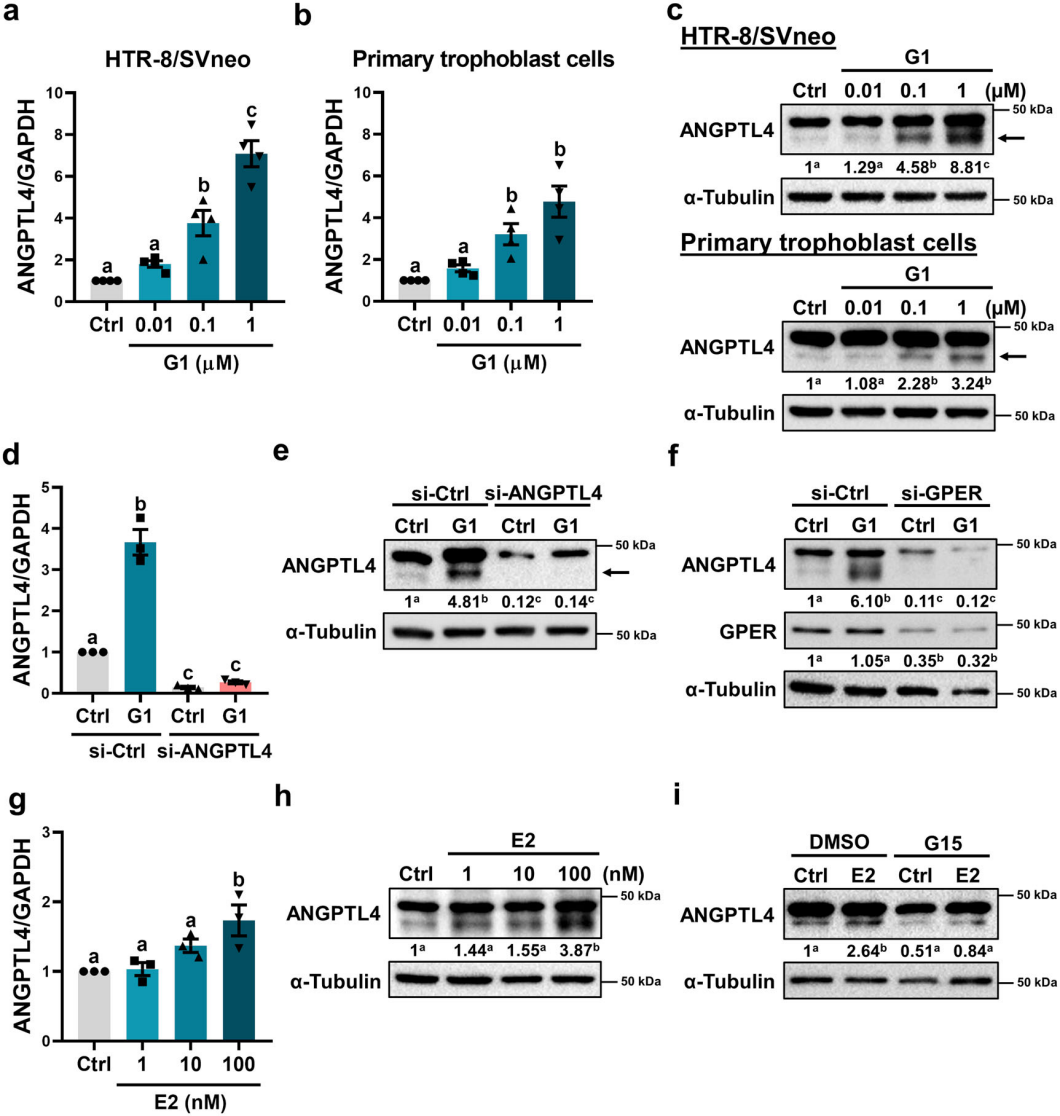
Activation of GPER stimulates ANGPTL4 expression in human trophoblast cells. **a**, **b** HTR-8/SVneo cells (**a**) and primary cultures of human trophoblast cells (**b**) were treated with different concentrations of G1 for 24 h, and ANGPTL4 mRNA levels were examined by RT-qPCR (*n* = 4). **c** HTR-8/SVneo cells and primary cultures of human trophoblast cells were treated with different concentrations of G1 for 24 h, and ANGPTL4 protein levels were examined by western blot (*n* = 4). The arrow indicates the ANGPTL4 band. **d**, **e** HTR-8/SVneo cells were transfected with 50 nM control siRNA (si-Ctrl) or ANGPTL4 siRNA (si-ANGPTL4) for 48 h and then treated with 0.1 µM G1 for 24 h. ANGPTL4 mRNA (**d**) and protein (**e**) levels were examined by RT-qPCR and western blot, respectively (*n* = 3). **f** HTR-8/SVneo cells were transfected with 50 nM control siRNA (si-Ctrl) or GPER siRNA (si-GPER) for 48 h and then treated with 0.1 µM G1 for 24 h. ANGPTL4 and GPER protein levels were examined by western blot (*n* = 3). **g**, **h** HTR-8/SVneo cells were treated with different concentrations of E2 for 24 h. ANGPTL4 mRNA (**g**) and protein (**h**) levels were examined by RT-qPCR and western blot, respectively (*n* = 3). **i** HTR-8/SVneo cells were pretreated with vehicle control (DMSO) or 1 µM G15 for 1 h and then exposed to 100 nM E2 for 24 h. ANGPTL4 protein levels were examined by western blot (*n* = 3). The RT-qPCR results are expressed as the mean ± SEM. Numbers under the western blots represent the densitometry quantifications. Values without a common letter are significantly different (*P* < 0.05).

To further confirm our observations derived from the RNA-seq results, the stimulatory effect of G1 on ANGPTL4 expression was tested in HTR-8/SVneo cells by treating cells with different concentrations of G1. RT-qPCR results showed that treatment of G1 induced ANGPTL4 mRNA levels in a dose-dependent manner (Fig. 2a). Similar results were obtained in primary cultures of human trophoblast cells (Fig. 2b). Western blot results also confirmed the stimulatory effect of G1 on ANGPTL4 protein levels (Fig. 2c). Given that multiple bands were detected by western blot analysis, a siRNA-mediated knockdown approach was applied to verify our results. As shown in Fig. 2d and e, transfection of ANGPTL4 siRNA downregulated basal and G1-induced ANGPTL4 mRNA and protein levels. Importantly, siRNA-mediated knockdown indicated the correct band of ANGPTL4 that was analyzed by western blot. In addition, GPER knockdown abolished the stimulatory effect of G1 on ANGPTL4 expression (Fig. 2f and Supplementary Fig. 4a). E2 treatment of HTR-8/SVneo cells also increased ANGPTL4 mRNA and protein levels; however, the effects were slightly lower than those induced by G1 (Fig. 2g, h). Moreover, E2-induced ANGPTL4 expression was attenuated by pretreatment with the GPER-selective antagonist G15^22^ (Fig. 2i and Supplementary Fig. 4b). Collectively, these results clearly indicate that ANGPTL4 is a target gene of GPER in human trophoblast cells.

### YAP is a downstream signaling mediator of GPER in human trophoblast cells

**Fig. 3:**
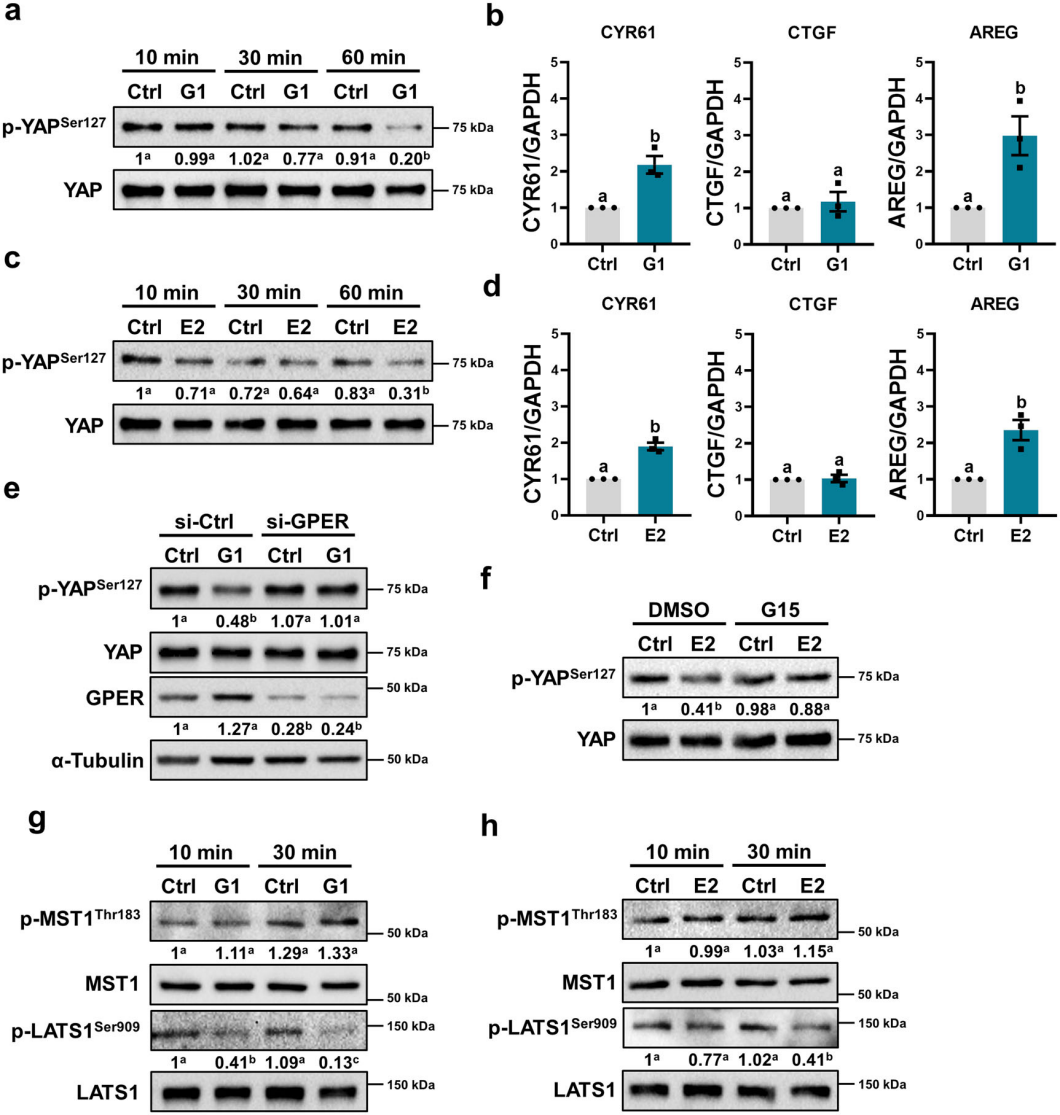
Activation of GPER activates YAP in human trophoblast cells. **a** HTR-8/SVneo cells were treated with 0.1 µM G1 for 10, 30, and 60 min. YAP phosphorylation levels at Ser127 (p-YAP^Ser127^) were examined by western blot (*n* = 3). **b** HTR-8/SVneo cells were treated with 0.1 µM G1 for 18 h. CYR61, CTGF, and AREG mRNA levels were examined by RT-qPCR (*n* = 3). **c** HTR-8/SVneo cells were treated with 100 nM E2 for 10, 30, and 60 min. YAP phosphorylation levels at Ser127 (p-YAP^Ser127^) were examined by western blot (*n* = 3). **d** HTR-8/SVneo cells were treated with 100 nM E2 for 18 h. CYR61, CTGF, and AREG mRNA levels were examined by RT-qPCR (*n* = 3). **e** HTR-8/SVneo cells were transfected with 50 nM control siRNA (si-Ctrl) or GPER siRNA (si-GPER) for 48 h and then treated with 0.1 µM G1 for 60 min. YAP phosphorylation levels at Ser127 (p-YAP^Ser127^) and GPER expression levels were examined by western blot (*n* = 3). **f** HTR-8/SVneo cells were pretreated with vehicle control (DMSO) or 1 µM G15 for 1 h and then exposed to 100 nM E2 for 60 min. YAP phosphorylation levels at Ser127 (p-YAP^Ser127^) were examined by western blot (*n* = 4). **g**, **h** HTR-8/SVneo cells were treated with 0.1 µM G1 (**g**) or 100 nM E2 (**h**) for 10 and 30 min. The phosphorylation levels of MST1 at Thr183 (p-MST1^Thr183^) and LATS1 at Ser909 (p-LATS1^Ser909^) were examined by western blot (*n* = 4). RT-qPCR results are expressed as the mean ± SEM. Numbers under the western blots represent the densitometry quantifications. Values without a common letter are significantly different (*P* < 0.05).

The Hippo pathway was first identified in *Drosophila* and is a critical regulator of organ size^23^. A recent study demonstrated that Yes-associated protein (YAP), the major downstream effector of the Hippo pathway, plays a pivotal role in placental development^24^. Phosphorylation of the YAP protein at Ser127 prevents its nuclear translocation and transcription coactivator function by promoting YAP binding to cytoplasmic 14-3-3 proteins^25^. To examine whether G1 activates YAP in human trophoblast cells, HTR-8/SVneo cells were treated with G1 for different time periods. G1 treatment decreased the phosphorylation levels of YAP at Ser127, indicating the activation of YAP (Fig. 3a). The expression levels of well-characterized YAP target genes were also examined. RT-qPCR results showed that cysteine-rich angiogenic inducer 61 (CYR61) and amphiregulin (AREG) mRNA levels were upregulated after G1 treatment (Fig. 3b). Similar results were obtained by treating HTR-8/SVneo cells with E2 (Fig. 3c, d). GPER knockdown abolished the stimulatory effect of G1 on the activation of YAP (Fig. 3e). In addition, E2-induced YAP activation was blocked by the inhibition of GPER (Fig. 3f). These results suggest that YAP may be an important intracellular mediator of GPER-mediated biological functions. We also explored the effect of G1 and E2 on the core upstream components of the Hippo pathway. Treatment of HTR-8/SVneo cells with G1 or E2 decreased large tumor suppressor 1 and 2 (LATS1/2) phosphorylation levels at Ser909. Unexpectedly, G1 or E2 treatment did not affect mammalian STE20-like protein kinase 1 (MST1) phosphorylation levels (Fig. 3g, h). RNA-seq results showed that the expression levels of major components of the Hippo pathway were not affected by G1 treatment (Supplementary Fig. 5).

### YAP mediates activation of GPER-induced ANGPTL4 expression in human trophoblast cells

**Fig. 4:**
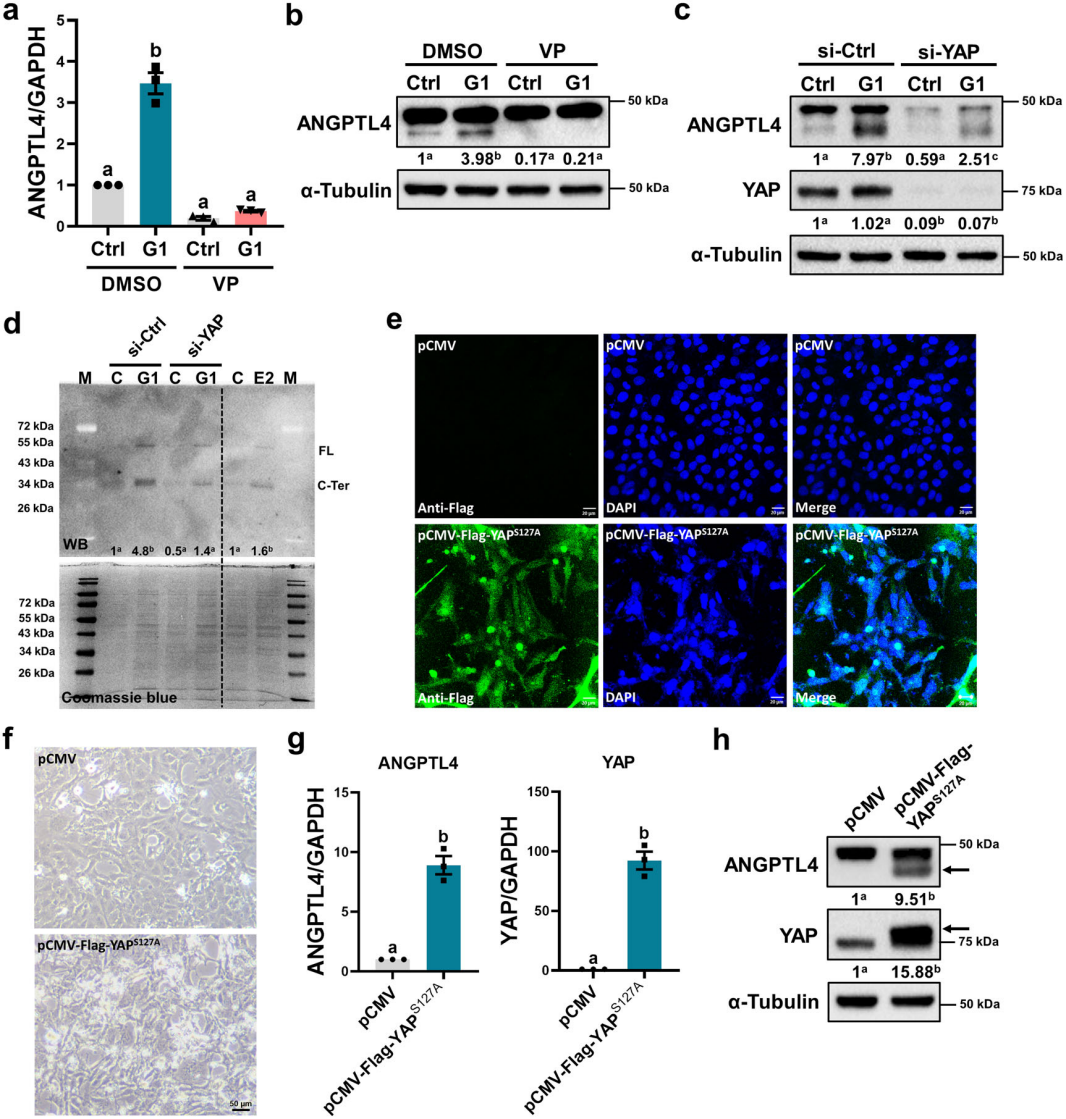
YAP mediates the activation of GPER-stimulated ANGPTL4 expression. **a**, **b** HTR-8/SVneo cells were pretreated with vehicle control (DMSO) or 5 µM verteporfin (VP) for 1 h and then exposed to 0.1 µM G1 for 24 h. ANGPTL4 mRNA (**a**) and protein (**b**) levels were examined by RT-qPCR and western blot, respectively (*n* = 3). **c** HTR-8/SVneo cells were transfected with 50 nM control siRNA (si-Ctrl) or YAP siRNA (si-YAP) for 48 h and then treated with 0.1 µM G1 for 24 h. ANGPTL4 and YAP protein levels were examined by western blot (*n* = 3). **d** ANGPTL4 protein levels in culture media of HTR-8/SVneo cells after treatments were examined by western blot (WB). The levels of total protein in the same sample set were examined by Coomassie blue staining (*n* = 3). **e**, **f** HTR-8/SVneo cells were transfected with 1 µg control vector (pCMV) or vector containing YAP^S127A^ cDNA (pCMV-Flag-YAP^S127A^) for 48 h. The expression of Flag-tag was examined by immunofluorescence staining (**e**), and the resultant morphology was microscopically examined (**f**). **g**, **h** HTR-8/SVneo cells were transfected with 1 µg control vector (pCMV) or vector containing YAP^S127A^ cDNA (pCMV-Flag-YAP^S127A^) for 48 h. The mRNA (**g**) and protein (**h**) levels of ANGPTL4 and YAP were examined by RT-qPCR and western blot, respectively (*n* = 4). The RT-qPCR results are expressed as the mean ± SEM. Numbers under the western blots represent the densitometry quantifications. Values without a common letter are significantly different (*P* < 0.05).

To further dissect the relative contribution of YAP in mediating GPER-stimulated ANGPTL4 expression in human trophoblast cells, verteporfin (VP), a small molecule of the YAP inhibitor, was used to block the function of YAP^26^. Pretreatment with VP inhibited the stimulatory effect of G1 on ANGPTL4 mRNA and protein levels (Fig. 4a, b). To further confirm the involvement of YAP in G1-induced ANGPTL4 expression and avoid off-target effects of the pharmacologic molecules, YAP endogenous expression was knocked down by transfection of HTR-8/SVneo cells with YAP-specific siRNA. As shown in Fig. 4c and Supplementary Fig. 6, YAP knockdown partially attenuated the induction of ANGPTL4 mRNA and protein levels by G1 treatment. In addition, we detected the secreted ANGPTL4 protein in culture media after G1 treatment. Upon secretion into the circulation, ANGPTL4 is cleaved into an N-terminal domain and a C-terminal fibrinogen-like domain^27^. Our ANGPTL4 antibody can detect both full-length protein and the C-terminal fragment. As shown in Fig. 4d, consistent with a previous study, western blot analysis detected two bands that represented full-length ANGPTL4 and the C-terminal fragment of ANGPTL4 in the culture medium^28^. Importantly, treatment with G1 or E2 induced ANGPTL4 production into the culture medium. In addition, the knockdown of YAP partially attenuated G1-induced ANGPTL4 production. The levels of total protein were examined by parallel experiments with the same sample set using Coomassie blue staining. To further strengthen our observation that YAP mediated ANGPTL4 expression, a gain-of-function approach was applied. To this end, a constitutively active form of YAP, the YAP serine 127 to alanine (S127A) mutant with a Flag-tag, was transfected into HTR-8/SVneo cells. Immunofluorescence staining of the Flag-tag confirmed the transfection efficiency and showed that overexpressed YAP^S127A^ was mainly located in the nucleus, and cytoplasmic expression was also observed (Fig. 4e). Interestingly, overexpression of YAP^S127A^ resulted in a more fibroblastic spindle shape (Fig. 4f) and increased mRNA levels of the YAP target genes CYR61 and AREG (Supplementary Fig. 7). Importantly, RT-qPCR and western blot results showed that ANGPTL4 mRNA and protein levels were upregulated by YAP^S127A^ overexpression (Fig. 4g, h). Taken together, these results indicate that GPER-stimulated ANGPTL4 is mediated by YAP in human trophoblast cells.

### ANGPTL4 mediates G1-stimulated human trophoblast cell invasion

**Fig. 5:**
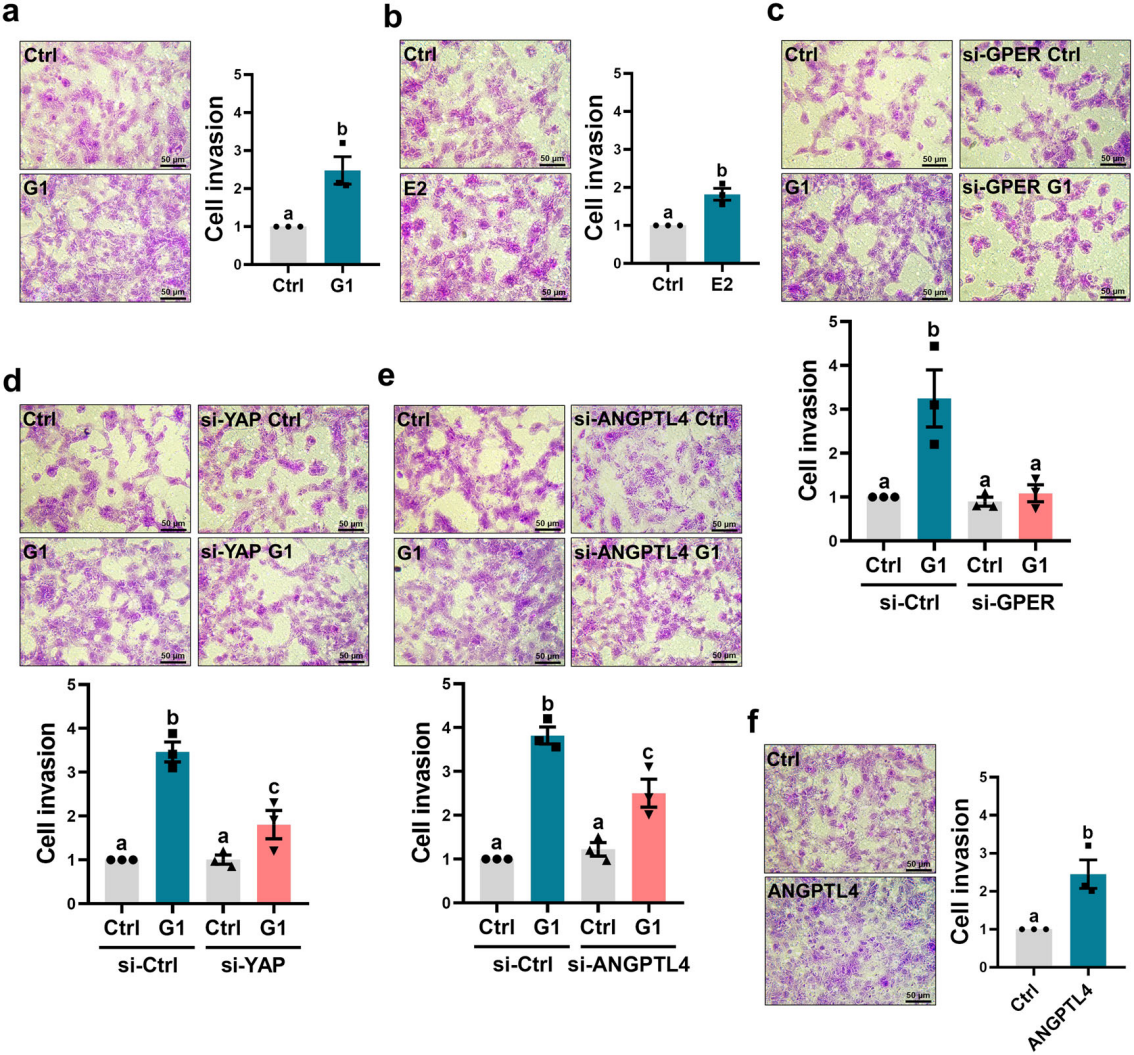
ANGPTL4 mediates the activation of GPER-stimulated human trophoblast cell invasion. **a**, **b** HTR-8/SVneo cells were treated with 0.1 µM G1 (**a**) or 100 nM E2 (**b**) and seeded onto Matrigel-coated transwell inserts. After 48 h of incubation, noninvading cells were wiped from the upper side of the filter, and the nuclei of the invading cells were stained with crystal violet (n = 3). **c**–**e** HTR-8/SVneo cells were transfected with 50 nM control siRNA (si-Ctrl), GPER siRNA (si-GPER) (**c**), YAP siRNA (si-YAP) (**d**), or ANGPTL4 siRNA (si-ANGPTL4) (**e**) for 48 h. The levels of cell invasiveness after treatments were examined by transwell invasion assay (*n* = 3). **f** HTR-8/SVneo cells were treated with 300 ng/mL human recombinant ANGPTL4, and cell invasiveness was examined by transwell invasion assay (*n* = 3). The invasion assay results are expressed as the mean ± SEM. Values without a common letter are significantly different (*P* < 0.05).

Trophoblast invasion is a tightly regulated process. Inadequate or incomplete trophoblast cell invasion leads to the development of PE. Transwell invasion assays showed that treatment with G1 or E2 for 48 h increased the invasiveness of HTR-8/SVneo cells (Fig. 5a, b). The stimulatory effect of G1 on cell invasion was abolished by the knockdown of GPER (Fig. 5c). Given the stimulatory effects of GPER on YAP activation and ANGPTL4 expression, we next examined the role of YAP in G1-stimulated cell invasion. As shown in Fig. 5d, knockdown of YAP partially inhibited G1-stimulated cell invasion. Similarly, G1-stimulated cell invasion was partially attenuated by ANGPTL4 knockdown (Fig. 5e). To further strengthen the pro-invasive role of ANGPTL4 in human trophoblast cells, the invasiveness of HTR-8/SVneo cells was examined after treatment with the human recombinant ANGPTL4 protein. As shown in Fig. 5f, treatment with human recombinant ANGPTL4 protein increased the invasiveness of HTR-8/SVneo cells. To confirm that the stimulatory effect of G1, E2, and ANGPTL4 on cell invasion was not due to differences in cell growth, cell proliferation after G1, E2, or ANGPTL4 treatment was examined using a trypan blue exclusion assay. As shown in Supplementary Fig. 8, G1 and E2 treatments slightly decreased HTR-8/SVneo cell proliferation only after 72 h in culture, whereas cell proliferation was not affected by human recombinant ANGPTL4 protein treatment.

### ANGPTL4 expression is downregulated in the serum and placenta of PE patients

**Fig. 6:**
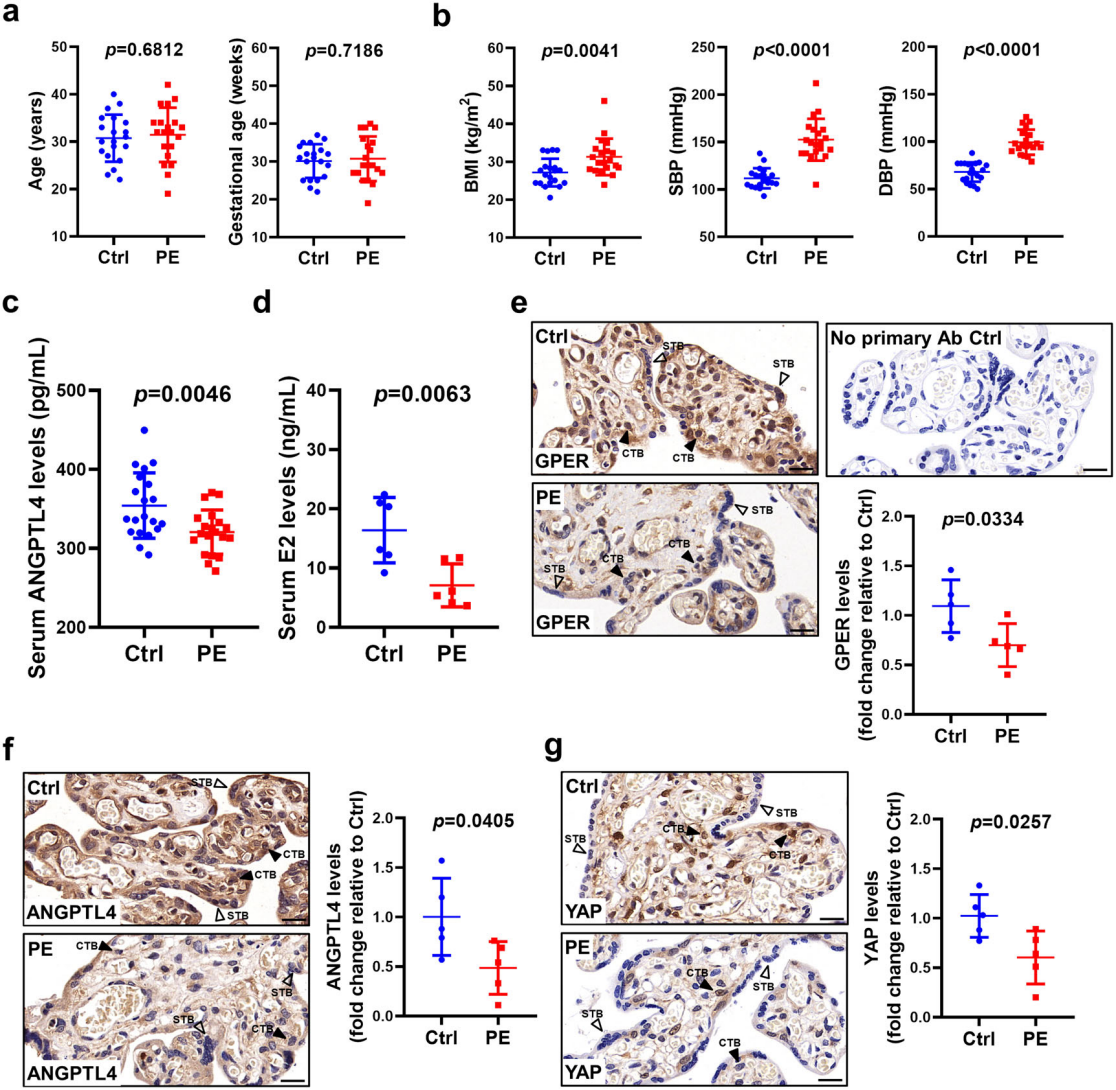
ANGPTL4 expression is downregulated in the serum and placenta of PE patients. **a**–**c** Serum samples were collected from 20 PE patients and 20 normal pregnant women of similar age and gestational age. The age and gestational age of the included patients are presented (**a**). BMI, systolic blood pressure (SBP), and diastolic blood pressure (DBP) were measured (**b**). ANGPTL4 serum levels were measured by ELISA (**c**). **d** E2 serum levels in patients in the second trimester of pregnancy were measured by ECLIA. **e**–**g** Representative images of immunohistochemical staining and quantification results for GPER (**e**), ANGPTL4 (**f**), and YAP (**g**) in the control and PE placenta. CTB cytotrophoblast cells, STB syncytiotrophoblast cells. Original magnification: ×400. The scale bar represents 50 μm. The results are expressed as the mean ± SD.

**Fig. 7:**
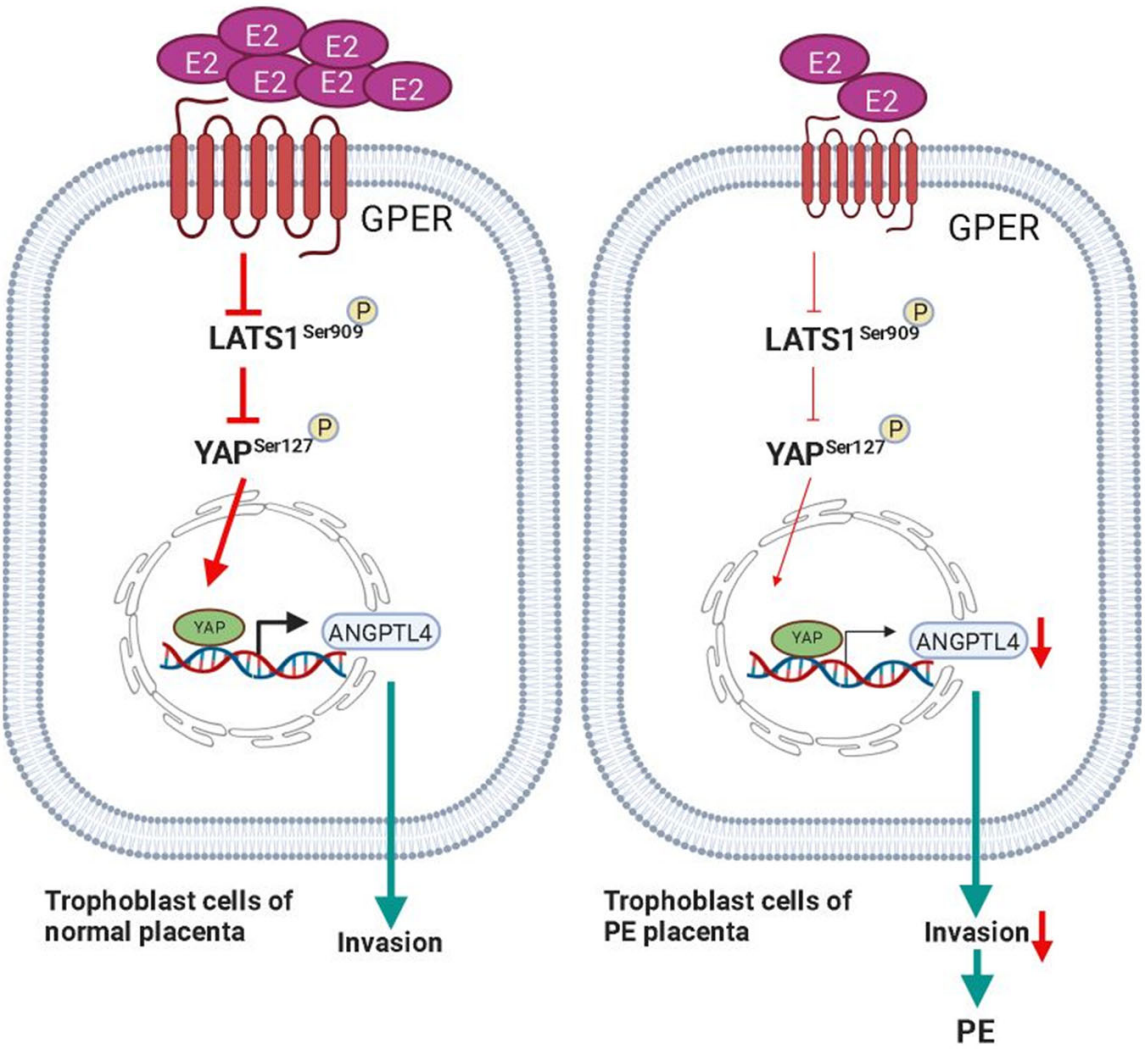
A schematic illustrating the signaling pathways for GPER-induced ANGPTL4 expression and the stimulation of human trophoblast cell invasion. Downregulation of E2 and GPER in PE leads to poor trophoblast cell invasion by decreasing ANGPTL4 expression.

ANGPTL4 is a secreted glycoprotein. Given the pro-invasive role of ANGPTL4 in human trophoblast cells, we were interested in comparing ANGPTL4 serum levels in patients with and without PE. To this end, ANGPTL4 serum levels were measured in 20 PE patients and 20 normal pregnant women of similar age and gestational age (Fig. 6a). As expected according to previous reports^10,24^, body mass index (BMI), systolic blood pressure (SBP), and diastolic blood pressure (DBP) were increased in PE patients compared with normal controls (Fig. 6b)^13,29^. Interestingly, ANGPTL4 serum levels were downregulated in PE patients (Fig. 6c). PE primarily affects women in the third trimester of pregnancy. Previous studies have shown that in the third trimester, serum E2 levels were lower in PE patients compared with normal pregnancy controls^14^. Here, we measured serum E2 levels in the second trimester of pregnancy. After follow-up, serum E2 levels in the second trimester were lower in patients who were later diagnosed with PE than in those who were not (Fig. 6d). Immunohistochemical (IHC) staining revealed that consistent with a previous study, the expression of GPER was detected in both CTB and STB cells^17^. In addition, the expression levels of GPER were downregulated in both cell types of term PE placenta (Fig. 6e). IHC results showed that ANGPTL4 was expressed in both CTB and STB cells. Similar to GPER, we found that downregulation of ANGPTL4 expression in both CTB and STB cells of PE placenta was observed (Fig. 6f). Consistent with the findings reported by a recent study^24^, YAP was expressed in CTB cells but not in STB cells. Here, we showed that the expression levels and nuclear localization of YAP were decreased in the CTB cells of the PE placenta (Fig. 6g). Taken together, these results demonstrate that ANGPTL4 expression was decreased in patients with PE, which could be attributed to the downregulation of GPER-mediated YAP activation (Fig. 7).

## Discussion

The major finding of this study is the identification of ANGPTL4, a multifunctional secreted glycoprotein, as a GPER target gene in human trophoblast cells. ANGPTL4 expression is under sensitive transcriptional control. Free fatty acid-activated PPARs transcriptionally stimulate ANGPTL4 expression via a functional PPAR-response element in the human ANGPTL4 gene^19,30^. Hippo pathway activation leads to stimulation of the serine/threonine kinases MST1/2, which phosphorylate the downstream kinases LATS1/2. Phosphorylated LATS1/2 subsequently phosphorylates YAP at Ser127, which results in its cytoplasmic retention and proteolytic degradation. In contrast, inhibition of the Hippo pathway dephosphorylates YAP, which prevents its export from the nucleus and promotes its transcriptional activities by interacting with the TEA domain protein (TEAD) family of transcription factors. To date, YAP has been shown to regulate numerous biological processes, including cell proliferation, invasion, and metabolism^31^. Our results showed that activation of GPER by G1 or E2 activated YAP by decreasing its phosphorylation levels at Ser127 in human trophoblast cells. Using loss- and gain-of-function approaches, we demonstrated that YAP mediated GPER-induced ANGPTL4 expression. A recent study using integrative genomic analysis demonstrated that in human ovarian cancer cells, ANGPTL4 is a direct target gene of Tafazzin (TAZ), which is another key transcriptional coactivator of the Hippo pathway^32^. These results together with our findings indicate that, in addition to PPARs, the Hippo pathway also plays an important role in the regulation of ANGPTL4 expression.

Within the Hippo pathway, LATS1/2-dependent phosphorylation is thought to be the most important event in the regulation of YAP activity because LATS1/2 knockout abolishes most of the YAP phosphorylation in response to many known upstream regulatory signals^33^. Interestingly, we found that GPER-induced YAP activation required LATS1 but not MST1. These results are similar to the findings obtained in human breast cancer cells^34^. In addition, few studies have reported that MST1/2 is not absolutely required for YAP regulation by a number of upstream signals. For example, MAPK kinase kinase kinases (MAP4K) have been identified to act in parallel with MST1/2 to phosphorylate and activate LATS1/2^33,35^. Sphingosine-1-phosphate activates YAP in an MST1/2-independent manner in human hepatocellular carcinoma cells^36^. YAP is phosphorylated in vivo on all five LATS1/2 phosphorylation consensus sites that are Ser61, Ser109, Ser127, Ser164, and Ser381. In addition, phosphorylation on several other residues including Thr63, Ser138, Ser289, Ser351, and Ser384 have also been identified^37^. To date, the roles of these phosphorylation sites in the regulation of YAP function remain largely unknown. It has been shown that YAP can be inhibited by two mechanisms that coordinately suppress YAP oncogenic activity. The Ser127 phosphorylation-mediated spatial regulation controls the nuclear-cytoplasmic shuttling. The Ser381 phosphorylation-mediated temporal regulation affects YAP protein stability and degradation^37^. In the present study, whether activation of GPER phosphorylates YAP at other sites in addition to Ser127 is unknown. Thus, investigation of whether G1 can affect YAP phosphorylation at different sites and subsequently contribute to the regulation of YAP function in human trophoblast cells will be of great interest.

YAP is expressed in human CTB cells where it plays a pivotal role in the maintenance of cell proliferation and stemness^24^. Our results showed that YAP expression and activity were decreased in PE trophoblast cells compared to control trophoblast cells. These results are similar to recent studies showing downregulated YAP and TAZ expression in the placenta of severe PE^38,39^. In HTR-8/SVneo cells, YAP stimulates cell invasion and decreases apoptosis but does not affect cell proliferation. In addition, YAP-stimulated trophoblast cell invasion is mediated by the YAP target gene caudal-related homeobox transcription factor 2 (CDX2)^38^. GPER plays an important role in cancer growth and metastasis^5^. Our transwell invasion assay showed that GPER and YAP were required for G1- or E2-induced trophoblast cell invasion. These results reveal that downregulation of GPER and YAP contributes to PE by impairing trophoblast cell invasion. Similar to GPER, aberrant ANGPTL4 expression is associated with tumorigenesis^19^. In this study, we provided evidence that ANGPTL4 mediated GPER-stimulated trophoblast cell invasion. It has been reported that ANGPTL4 promotes HTR-8/SVneo cell invasion by increasing the matrix metalloproteinase 2 and 9 (MMP2/9) and decreasing the tissue inhibitor of matrix metalloproteinase 1 and 2 (TIMP1/2) expression^40^. However, how ANGPTL4 triggers its biological effects remains unknown. In endothelial cells, ANGPTL4 binds directly to neuropilin 1 (NRP1) and NRP2 and regulates cell–cell junctions^41^. Whether the same is true in human trophoblast cells remains unclear and needs to be further defined. Interestingly, NPR1 expression is downregulated in PE trophoblast cells^42,43^. Collectively, these results support our conclusion that downregulation of ANGPTL4 results in insufficient trophoblast cell invasion in PE.

Our GO analysis results showed that activation of GPER upregulated genes that were enriched in the regulation of anion transport and lipid metabolism. Maintaining normal anion transport and lipid metabolism is required for placental development, and dysregulation of these events is associated with PE^44,45^. ANGPTL4 plays a vital role in lipid metabolism, as initially studied mainly for its role as a potent inhibitor of lipoprotein lipase (LPL), the enzyme responsible for the hydrolysis of circulating triglycerides into free fatty acids^46^. In PE, an increase in circulating free fatty acid concentrations above normal pregnancy levels has been observed^47^. A possible mechanism responsible for the increased free fatty acid levels could be the changes in LPL expression or activity. However, one study demonstrated that LPL is expressed in trophoblast cells and endothelial cells of the human placenta, but the protein levels of LPL and its endogenous enzyme activity did not vary between control and PE placentas^48^. Given the inhibitory role of ANGPTL4 in LPL, our study provides a potential mechanism whereby downregulation of ANGPTL4 expression in the placenta in PE contributes to the increased circulating free fatty acid levels by accentuating the function of LPL.

Consistent with two previous studies, we showed that GPER expression was downregulated in the placenta in PE^16,17^. However, the factors and underlying mechanisms that cause the downregulation of GPER in PE remain unknown. Previous studies have shown that the epidermal growth factor receptor (EGFR) ligands epidermal growth factor (EGF) and transforming growth factor-α (TGF-α) transactivate the GPER promoter and stimulate its expression in cancer cells^49,50^. Recently, we and other groups have shown that the invasiveness of HTR-8/SVneo cells is stimulated by treatment with EGFR ligands^51,52^. As EGF and TGF-α expression levels are reduced in the placenta of PE patients^53^, it is possible that these may contribute to the downregulation of GPER expression. In addition to transcriptional regulation, promoter methylation has been identified to affect GPER expression. In breast cancer, promoter hypermethylation is correlated with low GPER expression^54^. However, whether the pattern of GPER promoter methylation changes between normal and PE trophoblast cells is completely unknown and warrants further investigation.

The HTR-8/SVneo cell line was generated using first-trimester extravillous trophoblast cells infected with simian virus 40 large T antigen^55^. To date, the HTR-8/SVneo cell line remains the most commonly used cell model to study the biological functions of human trophoblast cells. In the present study, to make the experiments more technically feasible, particularly for those involving gene knockdowns or overexpression, HTR-8/SVneo cells were used as the experimental model. We are aware that this cell line can not fully represent the nature of normal human trophoblast cells and the results of the present study were derived mainly from the HTR-8/SVneo cells. Therefore, the stimulatory effect of GPER activation on ANGPTL4 expression and underlying mechanisms may need to be further confirmed in primary human trophoblast cells.

In summary, in this study, we identify ANGPTL4 as a GPER target gene in human trophoblast cells. We show that activation of GPER activates YAP and that is required for GPER-induced ANGPTL4 expression. Our results further demonstrate that GPER/YAP/ANGPTL4 stimulates trophoblast cell invasion. Furthermore, our clinical results reveal that GPER, YAP, and ANGPTL4 are downregulated in the placenta of PE. These results demonstrate that downregulation of GPER/YAP/ANGPTL4 can lead to poor trophoblast cell invasion, which subsequently contributes to the pathogenesis of PE. This study not only provides important insights into the regulation of ANGPTL4 expression but also increases the understanding of the biological roles of GPER/YAP/ANGPTL4 in the placenta.

## Methods

### RNA sequencing and analysis

HTR-8/SVneo cells were treated with vehicle control, dimethyl sulfoxide (DMSO), and 1 µM G1 for 24 h. After treatments, total RNA from each sample was extracted using TRIzol (Thermo Fisher Scientific) according to the manufacturer’s instructions. RNA purity was assessed using a NanoPhotometer spectrophotometer (IMPLEN), and RNA integrity was assessed using the RNA Nano 6000 Assay Kit of the Bioanalyzer 2100 system (Agilent Technologies). Sequencing libraries were generated using the NEBNext® Ultra™ II RNA Library Prep Kit from Illumina (NEB) according to the manufacturer’s protocol. RNA sequencing was conducted by Beijing Novogene Bioinformatics Technology using Illumina NovaSeq 6000 sequencing system (Illumina). To allow the comparison of gene expression profiles, values for each transcript were normalized and calculated as fragments per kilobase per million mapped reads (FPKM). Differentially expressed genes (DEGs) were calculated using the DEGseq package of R software^56^. Gene Ontology (GO) analysis and Kyoto Encyclopedia of Genes and Genomes (KEGG) pathway analysis were conducted to identify DEGs at the biologically functional levels. The Database for Annotation, Visualization, and Integrated Discovery (DAVID) was used to integrate functional genomic annotations. *P* < 0.05 was considered to indicate a statistically significant difference.

### Cell culture and reagents

The human trophoblast cell line HTR-8/SVneo was obtained from American Type Culture Collection through its official distributor in China, Beijing Zhongyuan Limited. HTR-8/SVneo is an SV40 large T antigen immortalized first-trimester short-lived extravillous trophoblast cell line^55^. Cells were cultured in a humidified atmosphere containing 5% CO_2_ and 95% air at 37 °C in Dulbecco’s modified Eagle’s medium/nutrient mixture F-12 Ham medium (DMEM/F-12; Gibco) supplemented with 10% charcoal/dextran-treated fetal bovine serum (FBS) (HyClone), 100 U/mL penicillin, and 100 μg/mL streptomycin sulfate (Boster). G1 and G15 were obtained from Cayman. 17β-estradiol was obtained from Sigma. Verteporfin was obtained from Tocris. Recombinant human ANGPTL4 was obtained from R&D systems. All antibodies used in this study are summarized in Supplemental Table 1.

### Primary trophoblast cell culture

The study received institutional approval (#2020-KY-164) and was carried out in accordance with the guidelines from the Zhengzhou University Research Ethics Board. Written informed consent was obtained from all of the subjects before participation in the study. Human trophoblast cells were isolated from first-trimester placental tissue explants as previously described^57,58^. Briefly, chorionic villi were washed with cold medium and finely minced. Fragments of the chorionic villi were allowed to adhere for 2–3 days, after which any nonadherent material was removed. These tissue explants were further cultured for 10–14 days, during which the culture medium was changed every 2 days. Trophoblast cells were separated from the villous explants by brief trypsin digestion. Cells were cultured in a humidified atmosphere containing 5% CO_2_ and 95% air at 37 °C in DMEM/F-12 supplemented with 10% charcoal/dextran-treated FBS, 100 U/mL penicillin, and 100 μg/mL streptomycin sulfate.

### Reverse transcription-quantitative real-time PCR (RT-qPCR)

Total RNA was extracted with the RNeasy Plus Mini Kit (QIAGEN) according to the manufacturer’s instructions. RNA (1 μg) was reverse-transcribed into first-strand cDNA with the iScript Reverse Transcription Kit (Bio-Rad Laboratories). Each 20-μL qPCR reaction contained 1X SYBR Green PCR Master Mix (Applied Biosystems), 60 ng of cDNA, and 250 nM of each specific primer. The primer sequences used for the present study are presented in Supplemental Table 2. qPCR was performed on an Applied Biosystems QuantStudio 12 K Flex system equipped with 96-well optical reaction plates. The specificity of each assay was validated by melting curve analysis and agarose gel electrophoresis of the PCR products. All of the RT-qPCR experiments were run in triplicate, and a mean value was used to determine the mRNA levels. Water and mRNA without RT were used as negative controls. Relative quantification of the mRNA levels was performed using the comparative Ct method with GAPDH as the reference gene and using the formula 2^–∆∆Ct^.

### Western blot analysis

Cells were lysed in cell lysis buffer (Cell Signaling Technology) supplemented with a protease inhibitor cocktail (Sigma). The protein concentration was analyzed by the BCA protein assay kit (Pierce, Thermo Scientific). Equal amounts of protein were separated by SDS-polyacrylamide gel electrophoresis and transferred onto PVDF membranes. After 1 h of blocking with 5% nonfat dry milk in Tris-buffered saline (TBS), the membranes were incubated overnight at 4 °C with primary antibodies diluted in 5% nonfat milk/TBS. Following primary antibody incubation, the membranes were incubated with appropriate HRP-conjugated secondary antibodies. Immunoreactive bands were detected using an enhanced chemiluminescent substrate (Bio-Rad Laboratories) and imaged with a ChemiDoc MP Imager (Bio-Rad Laboratories). Band intensities were quantified using the Scion Image software.

### Small interfering RNA (siRNA) transfection and overexpression

To knock down endogenous ANGPTL4, GPER or YAP, cells were transfected with 50 nM ON-TARGETplus SMARTpool siRNA targeting a specific gene (Dharmacon) using Lipofectamine RNAiMAX (Invitrogen). The ON-TARGETplus siCONTROL NON-TARGETING pool siRNA (Dharmacon) was used as the transfection control. To overexpress YAP, cells were transfected with 1 µg empty pCMV vector or vector encoding a full-length constitutively active form of YAP with N-terminal Flag-tag (Addgene) using Lipofectamine 3000 (Invitrogen).

### Invasion assay

Transwell cell culture inserts (8-µm pore size, 24 wells, BD Biosciences) were coated with 1 mg/mL growth factor-reduced Matrigel (BD Biosciences). Cells (1 × 10^5^ cells/insert) in DMEM/F-12 medium supplemented with 0.1% FBS were incubated for 48 h against a gradient of 10% FBS. Noninvasive cells were removed with a cotton swab from the upper side of the membrane. Cells that penetrated the membrane were fixed with cold methanol. Cells were stained with crystal violet (0.5%, Sigma) for 30 min and subsequently washed thoroughly with tap water. Each experiment was performed with triplicate inserts. In each insert, five microscopic fields were photographed under an optical microscope, and the cell number was counted manually.

### Detection of ANGPTL4 in culture media

HTR-8/SVneo cells were cultured in 6-well plates with 1.5 mL of culture medium. Cells were grown to full confluence and serum-starved in a medium without FBS for 24 h to induce quiescence before treatments. After treatments in serum-free medium, medium samples were collected and concentrated by using 10 K Amicon Ultra 2-mL Centrifugal Filters (MilliporeSigma). Equal volumes (40 µL) of concentrated medium were added to the sample loading buffer with β-mercaptoethanol. Two sets of the same sample set were prepared. Samples were heated to 100 °C for 5 min and then loaded onto two SDS-polyacrylamide gels. Proteins in one gel were transferred onto PVDF membranes. The ANGPTL4 protein was detected as mentioned above in the western blot section. The levels of total proteins in another gel were stained with Coomassie blue. The expression levels of both full-length and C-terminal ANGPTL4 were normalized to the total protein levels detected by Coomassie blue staining.

### Measurement of ANGPTL4 and 17β-estradiol

ANGPTL4 levels in human serum samples were measured using an enzyme-linked immunosorbent assay (ELISA). The Human ANGPTL4 ELISA Kit (#EHANGPTL4, Thermo Fisher Scientific) was used in accordance with the manufacturer’s protocol. The interassay CV and intraassay CV for ANGPTL4 ELISA were <12% and <10%, respectively. The analytical sensitivity of ANGPTL4 ELISA was 20 pg/mL. 17β-estradiol levels in human serum samples were measured using an electrochemiluminescence immunoassay (ECLIA). An Elecsys Estradiol III Kit (#06656021190, Roche) was used in accordance with the manufacturer’s protocol.

### Immunofluorescence staining

Cells were cultured on coverslips, fixed with 4% buffered paraformaldehyde, and then permeabilized with 0.1% Triton X-100 in phosphate-buffered saline (PBS). Cells were blocked with Dako Protein Block (Dako) for 1 h and incubated with antibody to Flag (Sigma) diluted in Dako Protein Block. Alexa 488-labeled donkey anti-mouse was used as a secondary antibody. Cells were counterstained with DAPI, rinsed with PBS, mounted with Gelvatol, and examined using a ZEISS confocal microscope LSM700.

### Immunohistochemistry

The study received institutional approval (#2020-KY-164) and was carried out in accordance with the guidelines from the Zhengzhou University Research Ethics Board. Written informed consent was obtained from all of the subjects before participation in the study. Paraffin-embedded sections (5 μm) obtained from three control and three PE placentas were deparaffinized and rehydrated. Antigen retrieval was conducted by boiling sections in sodium citrate buffer (pH 6.0) for 8 min. Endogenous peroxidase activity was blocked by incubating sections of 3% hydrogen peroxide solution at room temperature for 10 min. After 1 h of blocking with 3% bovine serum albumin in PBS, sections were incubated with specific primary antibodies overnight at 4 °C. Following primary antibody incubation, the sections were incubated with HRP-conjugated secondary antibody. Sections were developed using the Peroxidase/DAB Dako REAL EnVision Detection System (Dako) and counterstained with hematoxylin. Negative control in the absence of a primary antibody was performed in parallel. Five areas were randomly selected from each section of the individual patient and the integrated optical density values were measured by the Image-Pro Plus 6.0 software. The quantification results were normalized with means of controls.

### Statistics and reproducibility

At least three independent experiments performed on separate cell passages were conducted to achieve the biological replicates. The results are presented as the mean ± SEM or mean ± SD. All statistical analyses were analyzed by PRISM software. For experiments involving only two groups, data were analyzed by *t* test. Multiple comparisons were analyzed using one-way ANOVA followed by Tukey’s multiple comparison test. A significant difference was defined as *P* < 0.05.

### Reporting summary

Further information on research design is available in the Nature Research Reporting Summary linked to this article.

## Supplementary information

Supplementary Information

Supplementary Data 1

Supplementary Data 2

Reporting Summary

## Data Availability

RNA-seq data have been deposited in the NCBI Gene Expression Omnibus database under accession number GSE183879. The data that support the findings of this study are available within the article and its Supplementary Information and Supplementary Data files. Uncropped western blots are available in Supplementary Fig 9. Information on all differentially expressed genes is available in Supplementary Data 1. The source data underlying all graphs and charts are provided as Supplementary Data 2.
