# Supplementary material for: G protein-coupled estrogen receptor stimulates human trophoblast cell invasion via YAP-mediated ANGPTL4 expression
Source: Commun Biol. 2021 Nov 12;4:1285. doi: 10.1038/s42003-021-02816-5 (PMC8589964; doi:10.1038/s42003-021-02816-5)
Supplement: Supplementary file 1 — Supplementary Information [file 42003_2021_2816_MOESM1_ESM.pdf]

Supplementary Table 1

Name of Antibody	Manufacturer and catalog #	Species raised in; monoclonal or polyclonal	Applications	Dilution used
ANGPTL4	abcam (ab206420)	Polyclonal rabbit Ab	WB	2000x
ANGPTL4	Invitrogen (40-9800)	Polyclonal rabbit Ab	IHC	50x
GPER	abcam (ab154069)	Polyclonal rabbit Ab	WB/IHC	1000x/100x
Phospho-YAP (Ser127)	Cell Signaling Technology (13008)	Monoclonal rabbit Ab	WB	1000x
YAP	Cell Signaling Technology (12395)	Monoclonal mouse Ab	WB/IHC	2000x/100x
Phospho-LATS1 (Ser909)	Cell Signaling Technology (9157)	Polyclonal rabbit Ab	WB	1000x
LATS1	Cell Signaling Technology (3477)	Molyclonal rabbit Ab	WB	1000x
Phospho-MST1 (Thr183)	Cell Signaling Technology (49332)	Molyclonal rabbit Ab	WB	1000x
MST1	Cell Signaling Technology (14946)	Molyclonal rabbit Ab	WB	1000x
α-Tubulin	Santa Cruz (sc-23948)	Monoclonal mouse Ab	WB	5000x
Flag	Sigma (F1804)	Monoclonal mouse Ab	IF	100x

Supplementary Table 2

Name of Gene	Forward	Reverse
ANGPTL4	CCTCTCCGTACCCTTCTCCA	AGTACTGGCCGTTGAGGTTG
GPER	CTCTTCCCCATCGGCTTTGT	TACAGGTCGGGGATGGTCAT
CYR61	CCAGTGTACAGCAGCCTGAA	ACTTGGGCCGGTATTTCTTC
CTGF	GCGTGTGCACCGCCAAAGAT	CAGGGCTGGGCAGACGAACG
AREG	GAGCACCTGGAAGCAGTAACA	GGCTGCTAATGCAATTTTTGA
YAP	CCCGACTCCTTCTTCAAGC	GAGAAACAGCTCCCAACTGC
GAPDH	GAGTCAACGGATTTGGTCGT	GACAAGCTTCCCGTTCTCAG

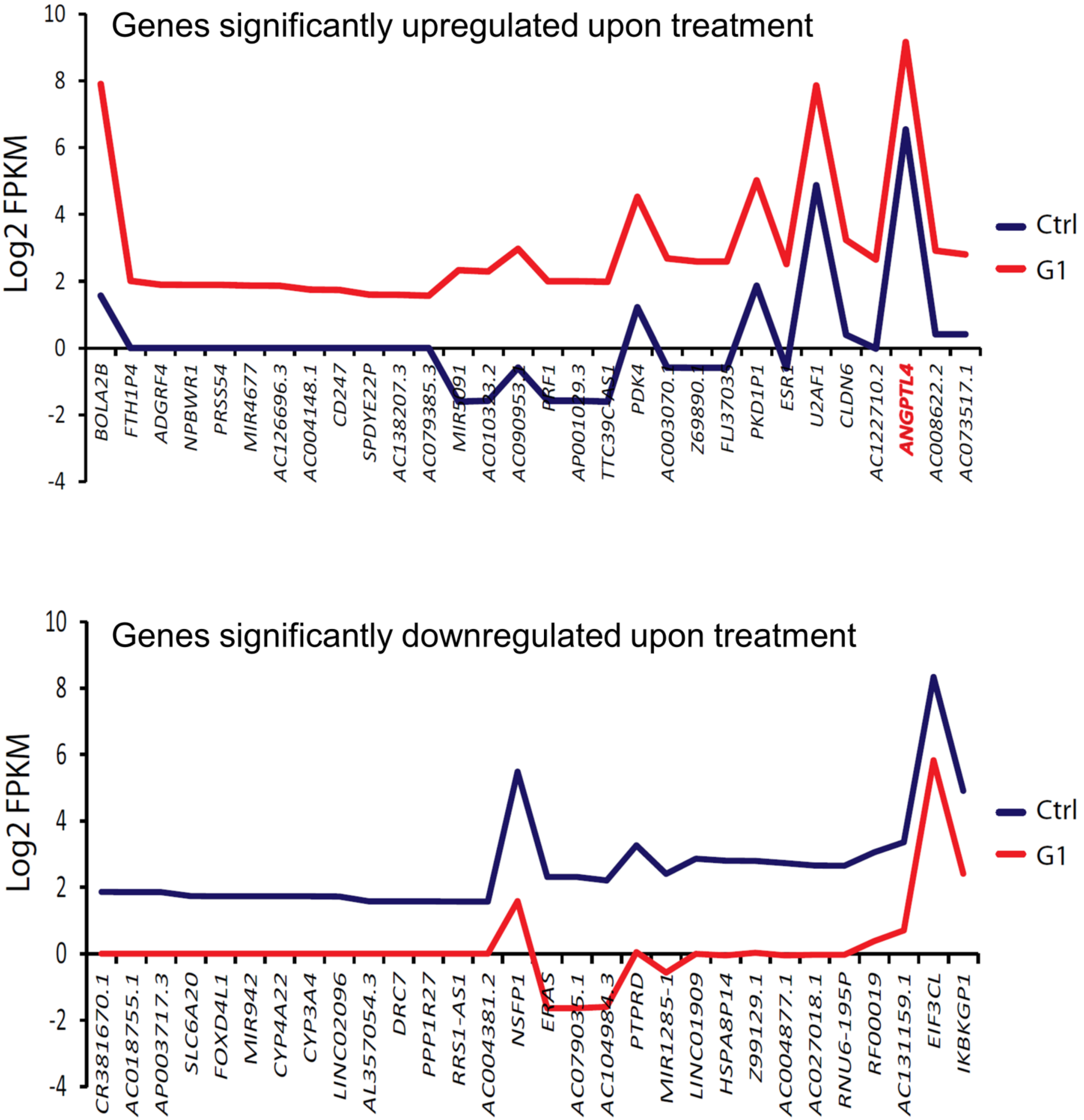

Supplementary Figure 1. Relative expression levels (log2 FPKM values) of the top 30 significantly altered genes regulated by G1 treatment.

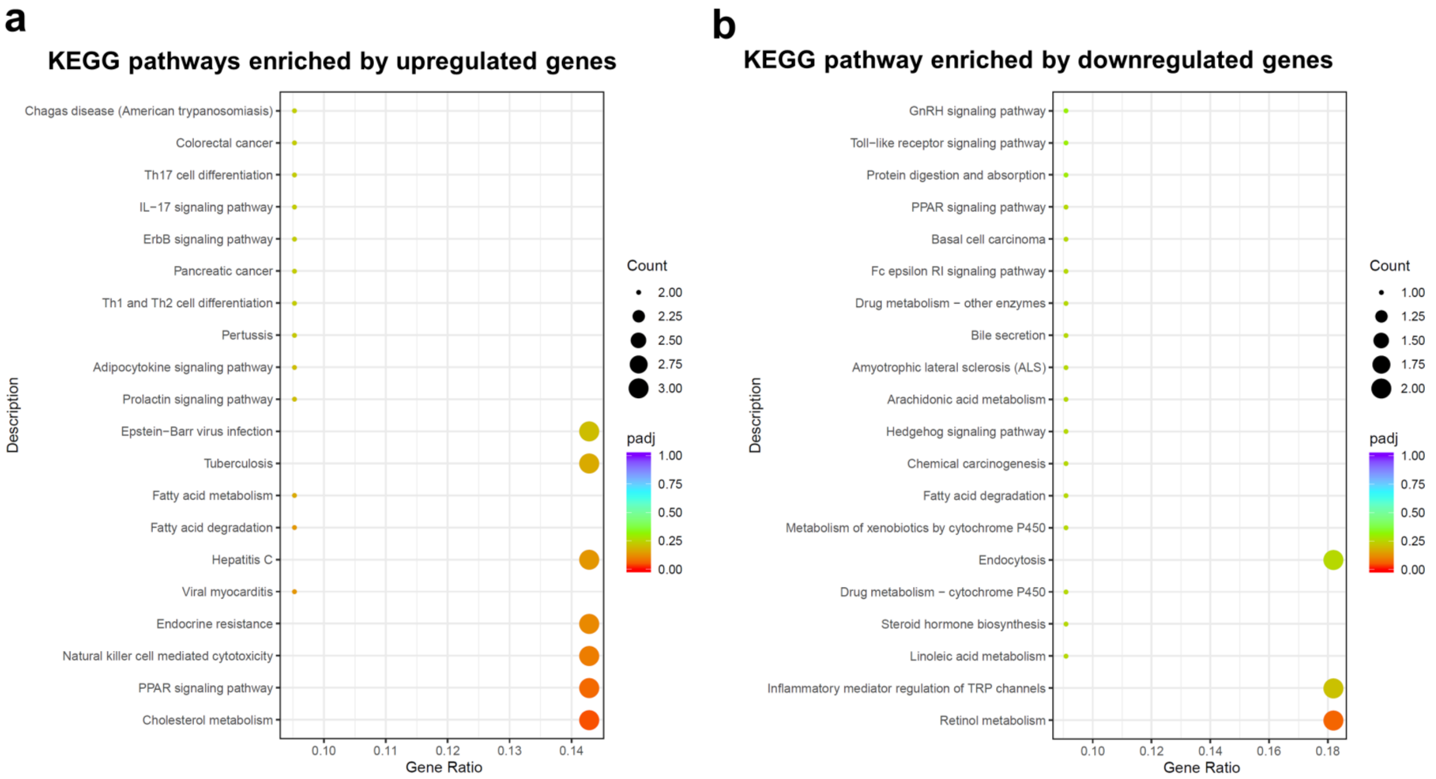

Supplementary Figure 2. KEGG signaling pathway analysis of DEGs.

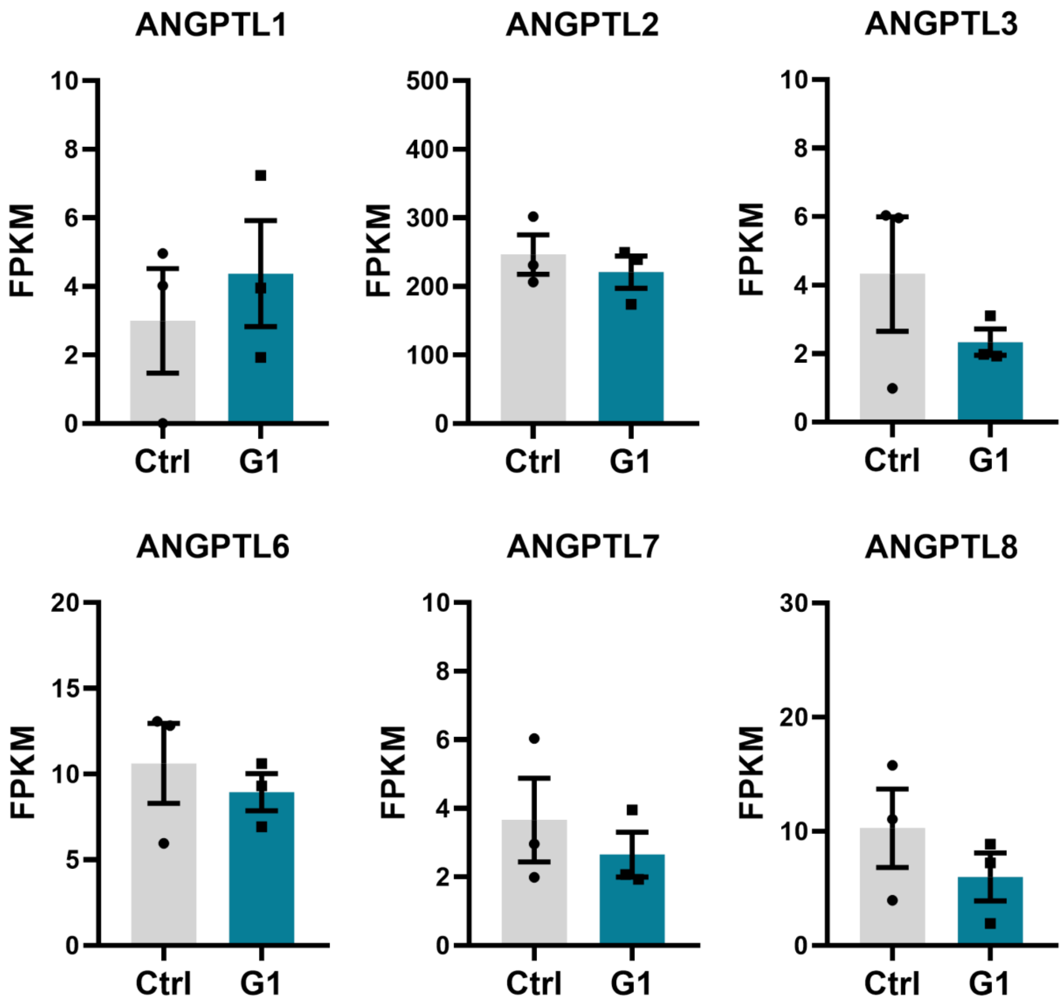

Supplementary Figure 3. RNA-seq results of ANGPTLs levels.

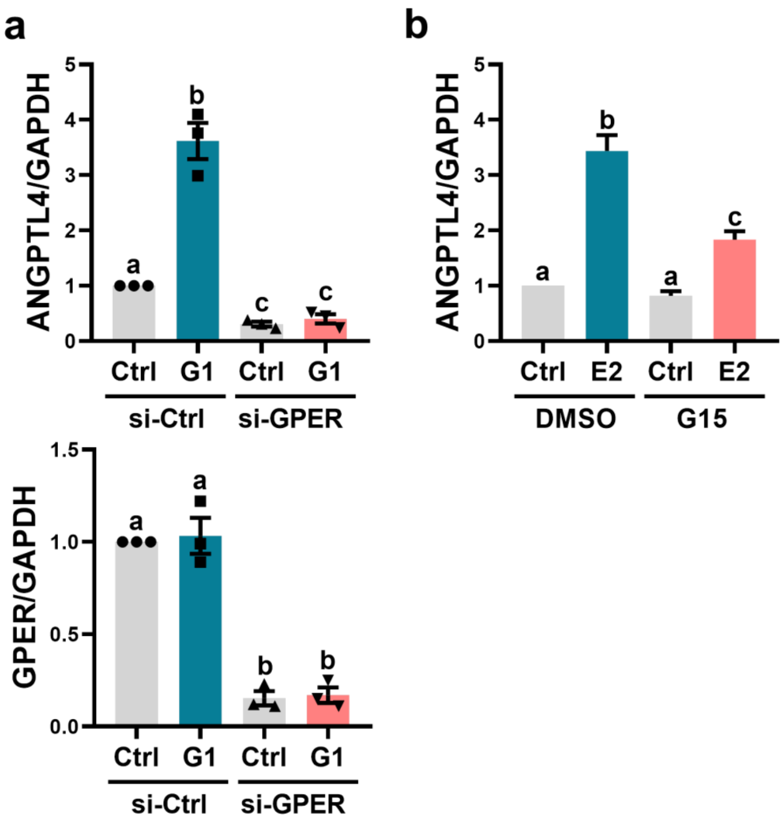

Supplementary Figure 4. Activation of GPER stimulates ANGPTL4 expression in human trophoblast cells. a, HTR-8/SVneo cells were transfected with 50 nM control siRNA (si-Ctrl) or GPER siRNA (si-GPER) for 48 h and then treated with 0.1 μM G1 for 24 h. ANGPTL4 and GPER mRNA levels were examined by RT-qPCR (n=3). **b,** HTR-8/SVneo cells were pretreated with vehicle control (DMSO) or 1 μM G15 for 1 h and then exposed to 100 nM E2 for 24 h. ANGPTL4 mRNA levels were examined by RT-qPCR (n=3). RT-qPCR results are expressed as the mean ± SEM. Values without a common letter are significantly different (p<0.05).

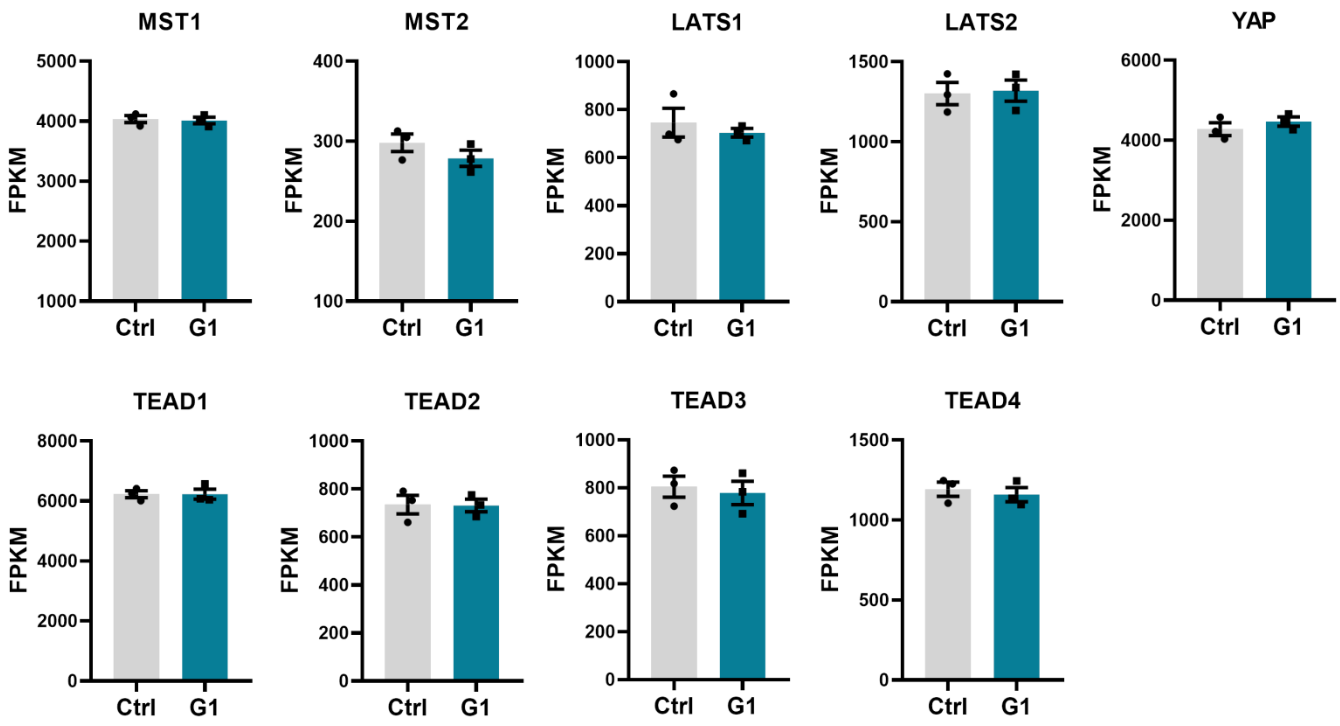

Supplementary Figure 5. RNA-seq results of the levels of Hippo pathway components.

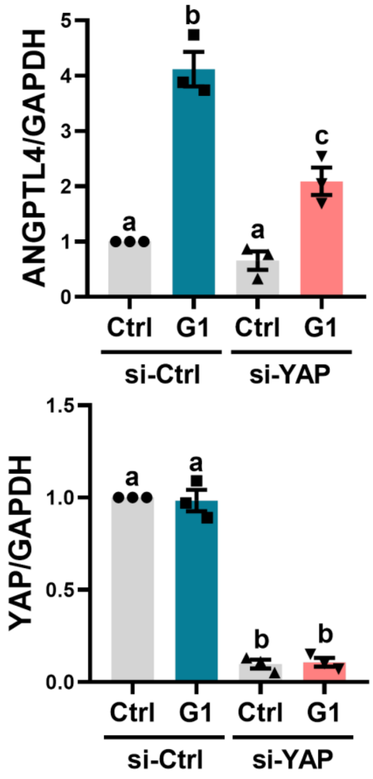

Supplementary Figure 6. YAP mediates the activation of GPER-induced ANGPTL4 expression. HTR-8/SVneo cells were transfected with 50 nM control siRNA (si-Ctrl) or YAP siRNA (si-YAP) for 48 h and then treated with 0.1 μM G1 for 24 h. ANGPTL4 and YAP mRNA levels were examined by RT-qPCR (n=3). RT-qPCR results are expressed as the mean ± SEM. Values without a common letter are significantly different (p<0.05).

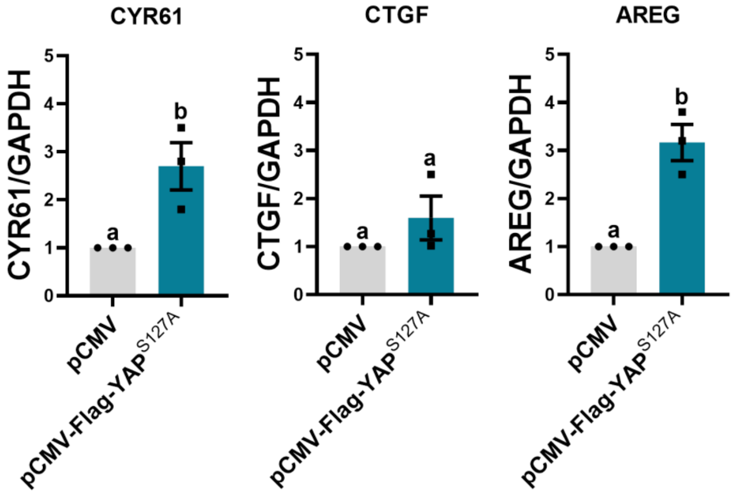

Supplementary Figure 7. Overexpression of YAP induces CYR61 and AREG expression. HTR-8/SVneo cells were transfected with 1 µg control vector (pCMV) or vector containing YAP^{S127A} cDNA (pCMV-Flag-YAP^{S127A}) for 48 h. CYR61, CTGF, and AREG mRNA levels were examined by RT-qPCR (n=3). RT-qPCR results are expressed as the mean ± SEM. Values without a common letter are significantly different (p<0.05).

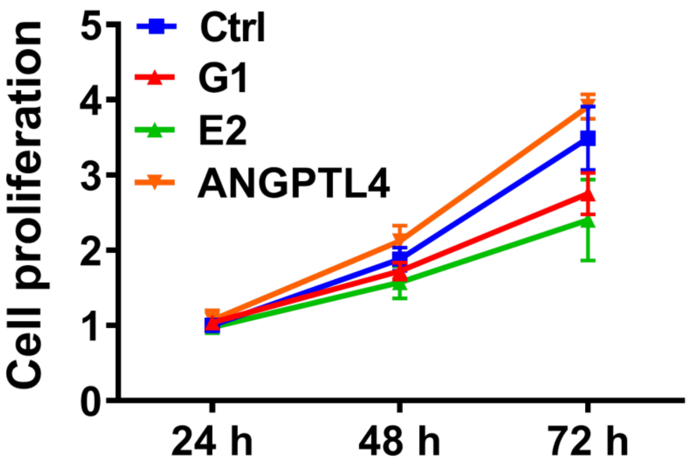

Supplementary Figure 8. The effects of G1, E2, and ANGPTL4 treatment on cell proliferation. HTR-8/SVneo cells were treated with 0.1 µM G1, 100 nM E2, or 300 ng/mL human recombinant ANGPTL4 every 24 h and cultured for up to 72 h. Cell numbers were measured by trypan blue exclusion assay (n=3). The results are expressed as the mean ± SEM.

Unedited blot for Figure 2b

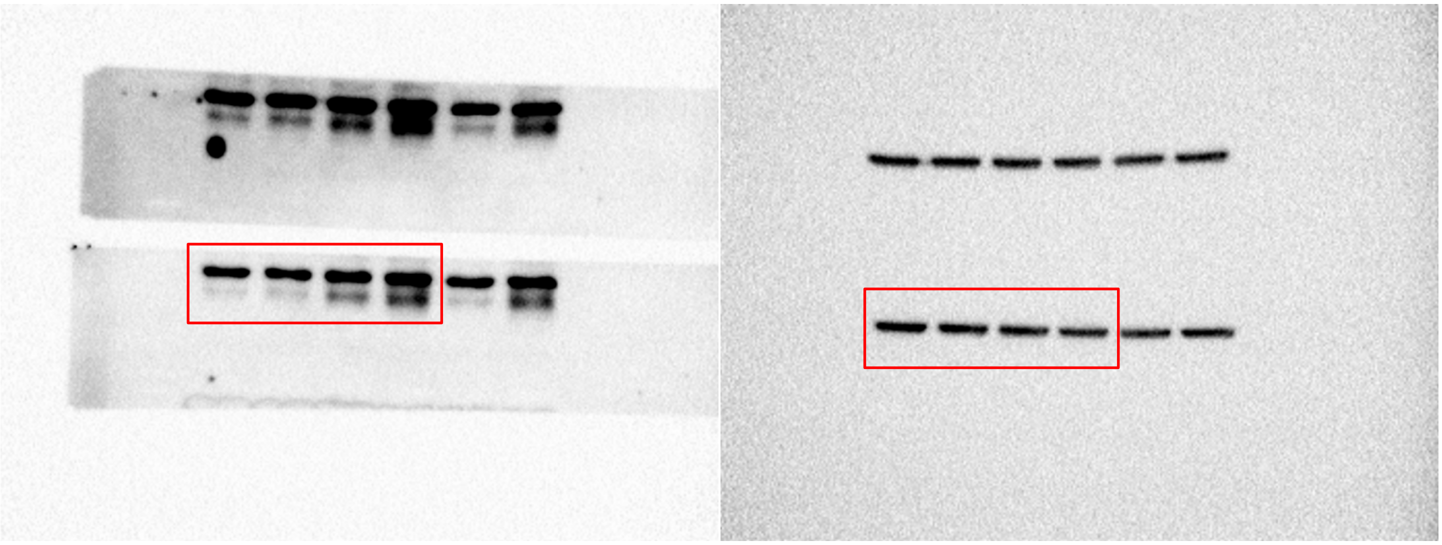

Unedited blot for Figure 2b

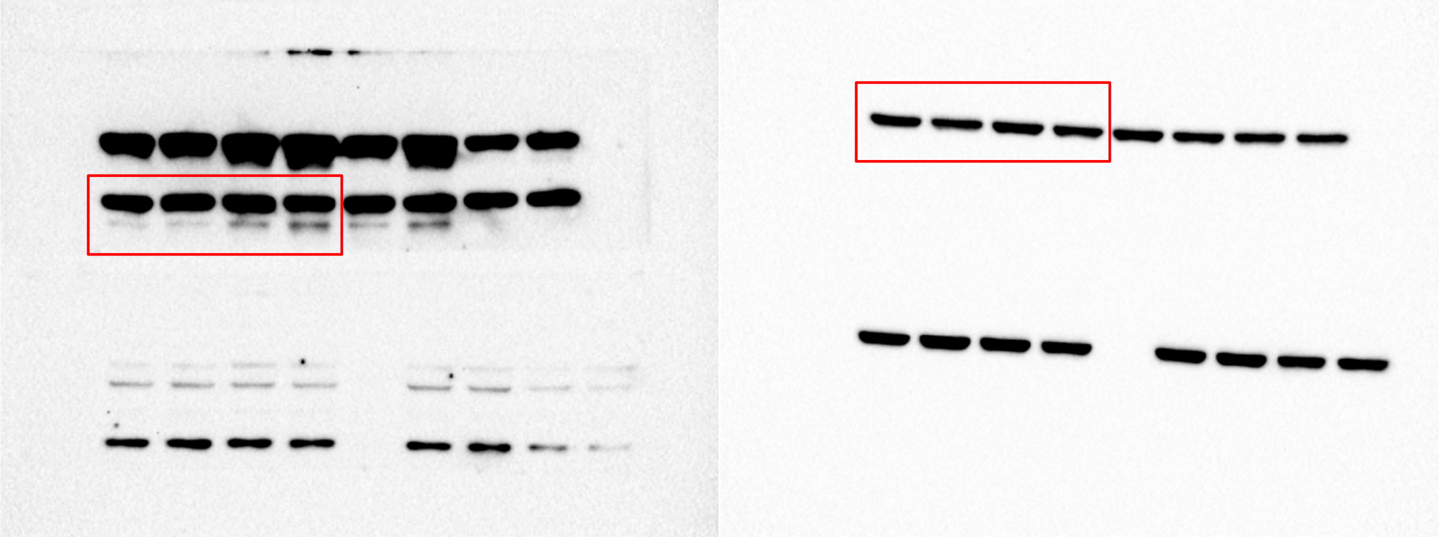

Unedited blot for Figure 2e

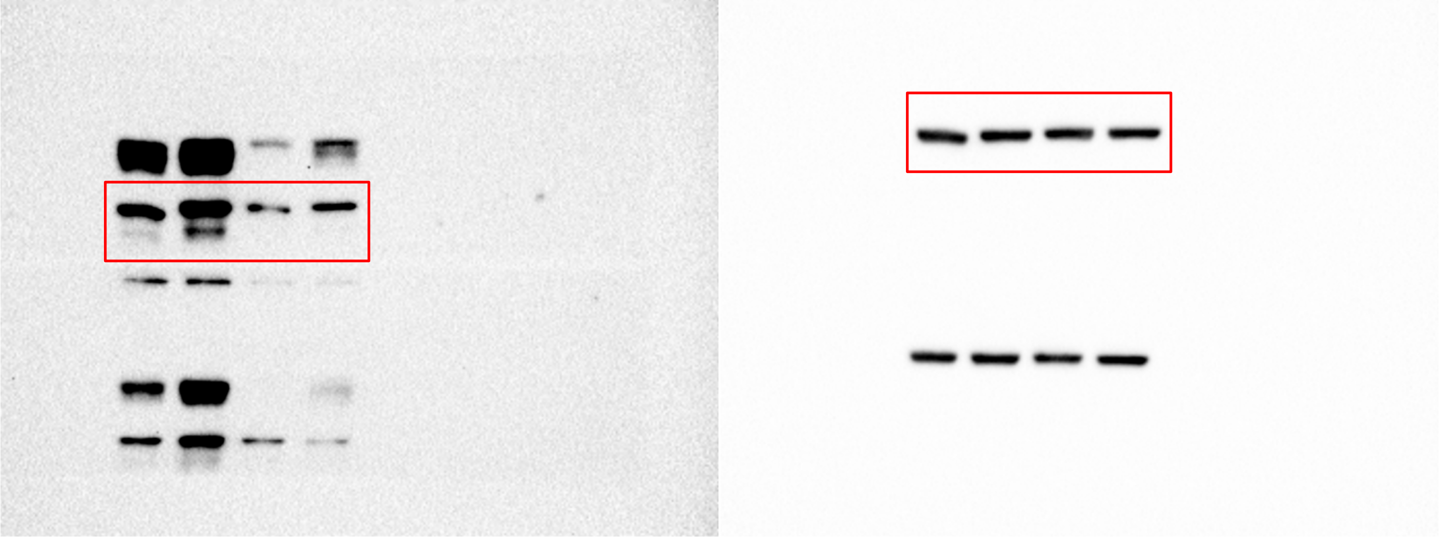

Unedited blot for Figure 2f

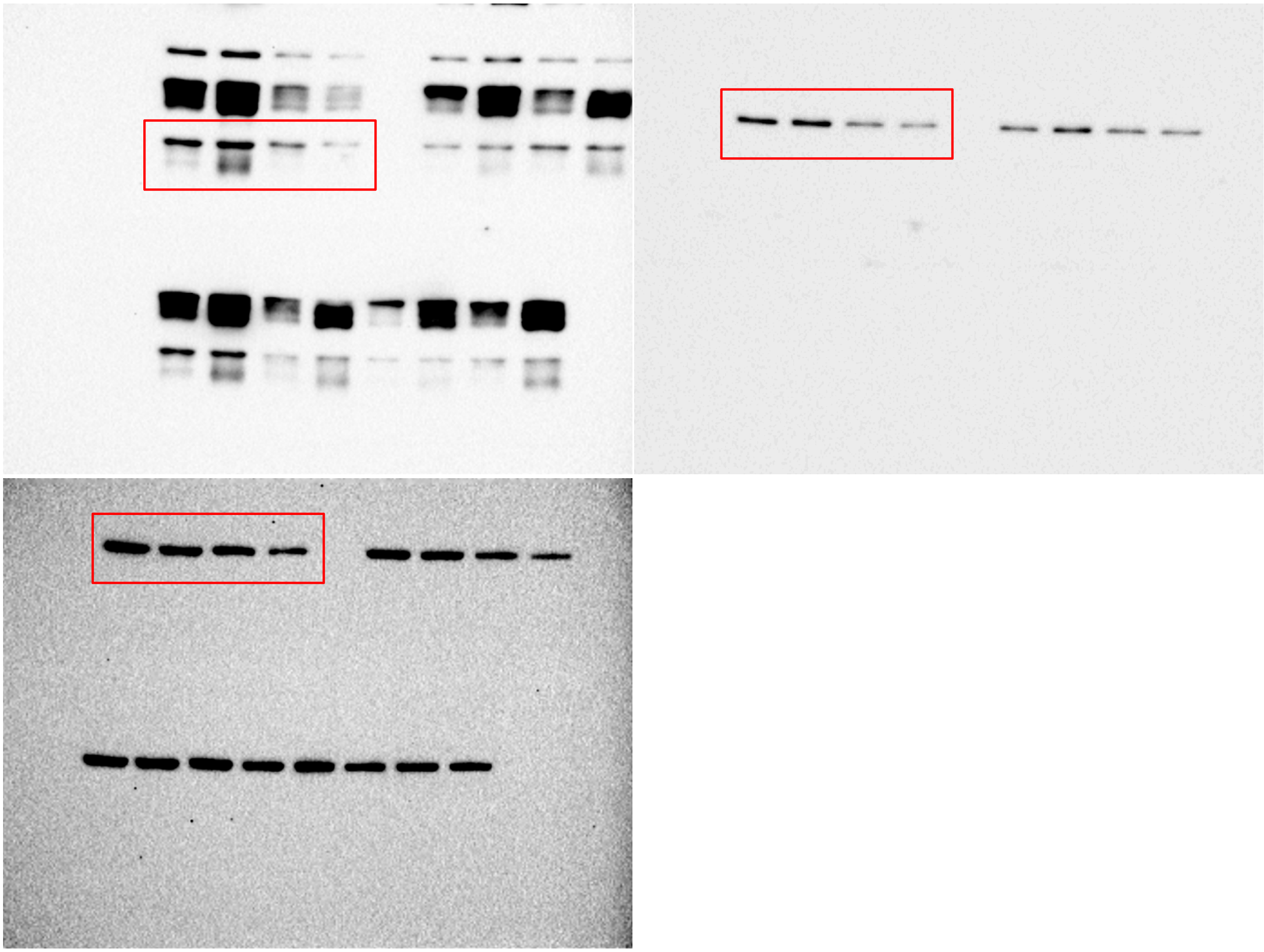

Unedited blot for Figure 2h

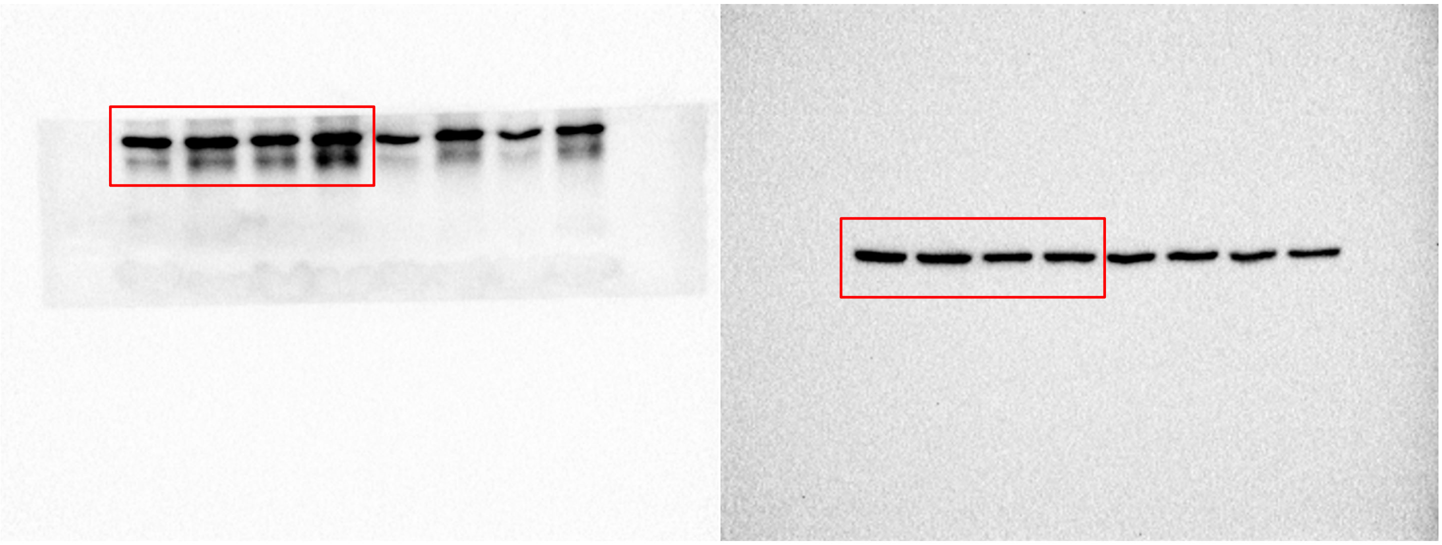

Unedited blot for Figure 2i

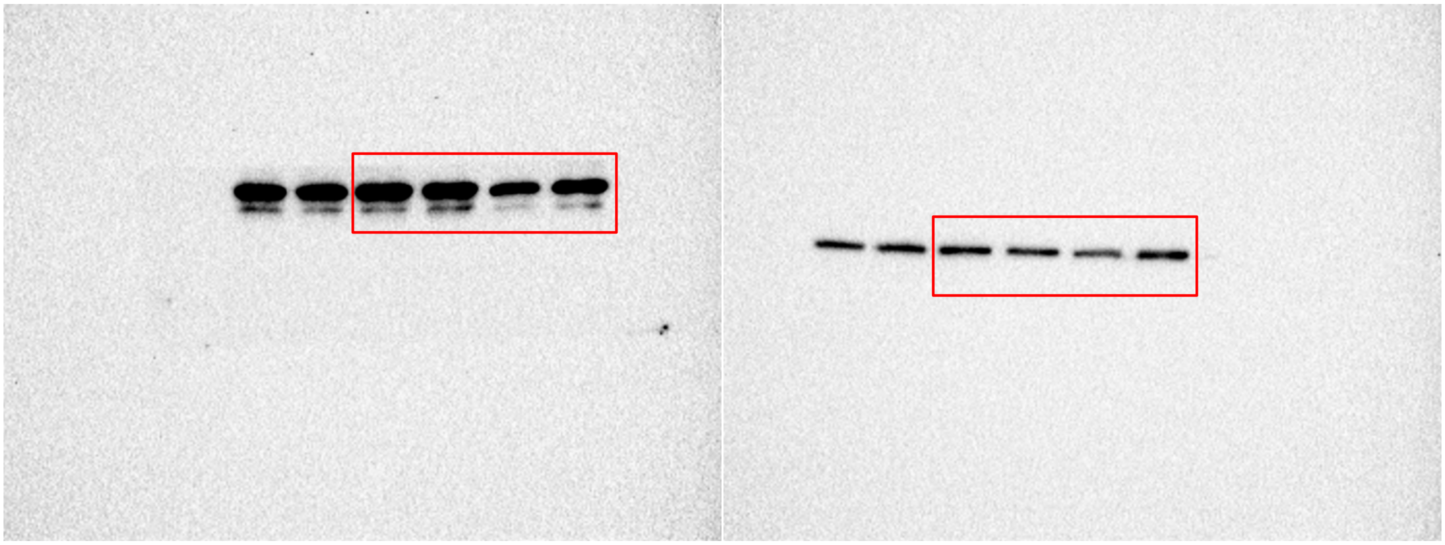

Unedited blot for Figure 3a

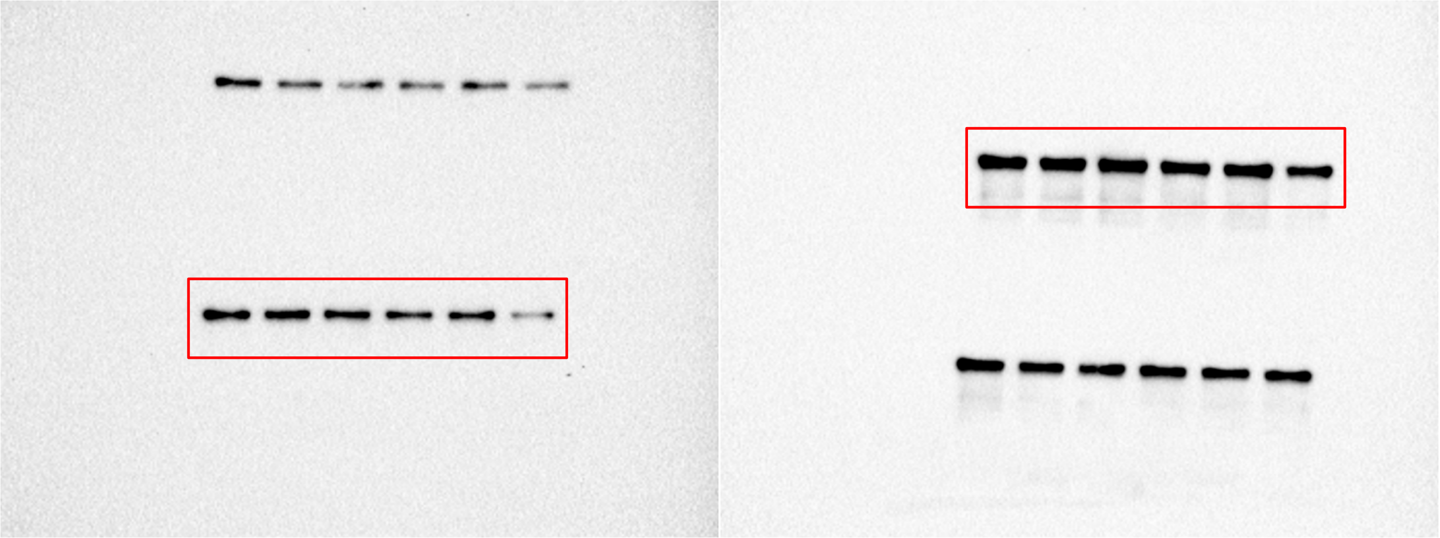

Unedited blot for Figure 3a

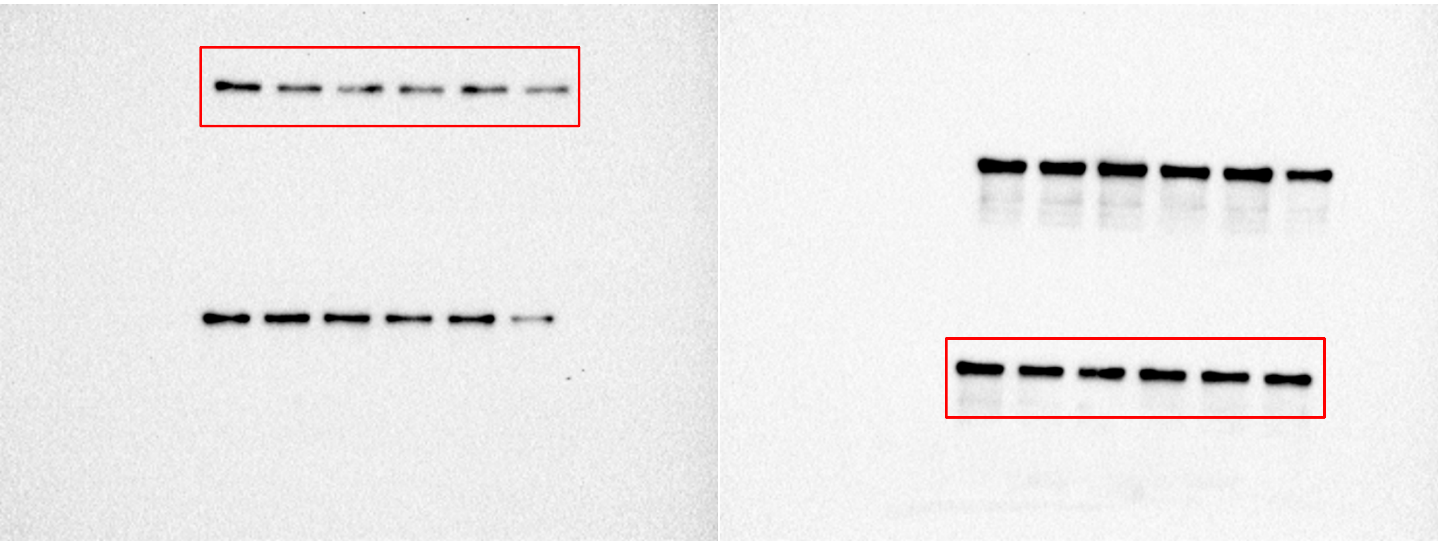

Unedited blot for Figure 3e

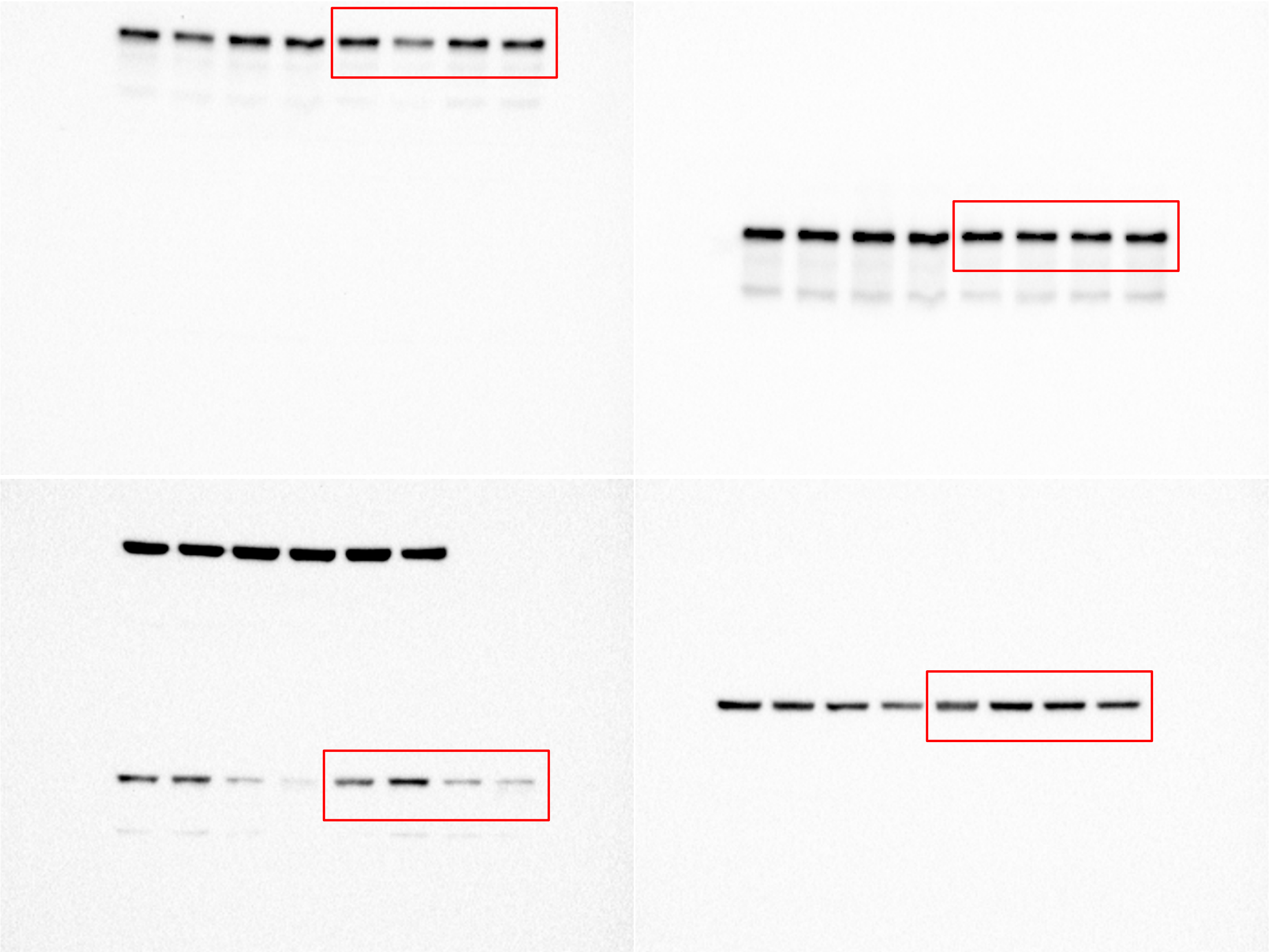

Unedited blot for Figure 3f

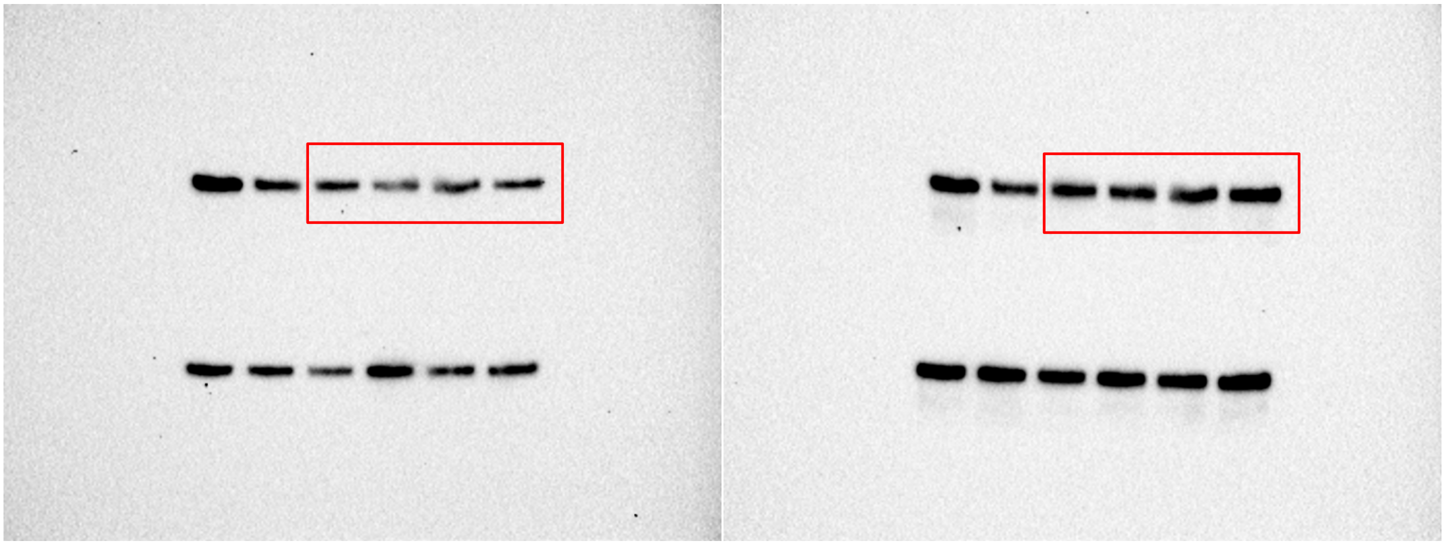

Unedited blot for Figure 3g

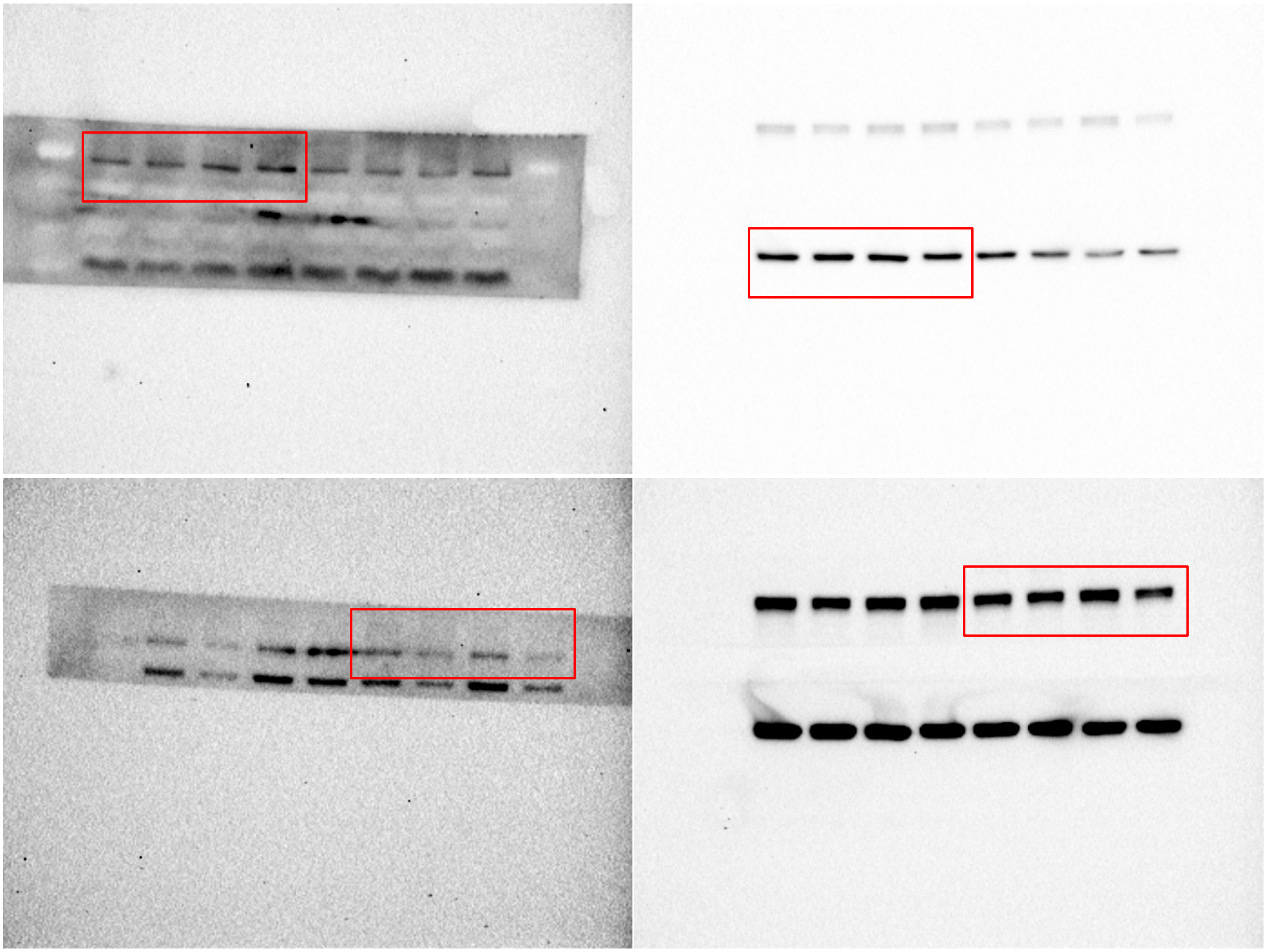

Unedited blot for Figure 3h

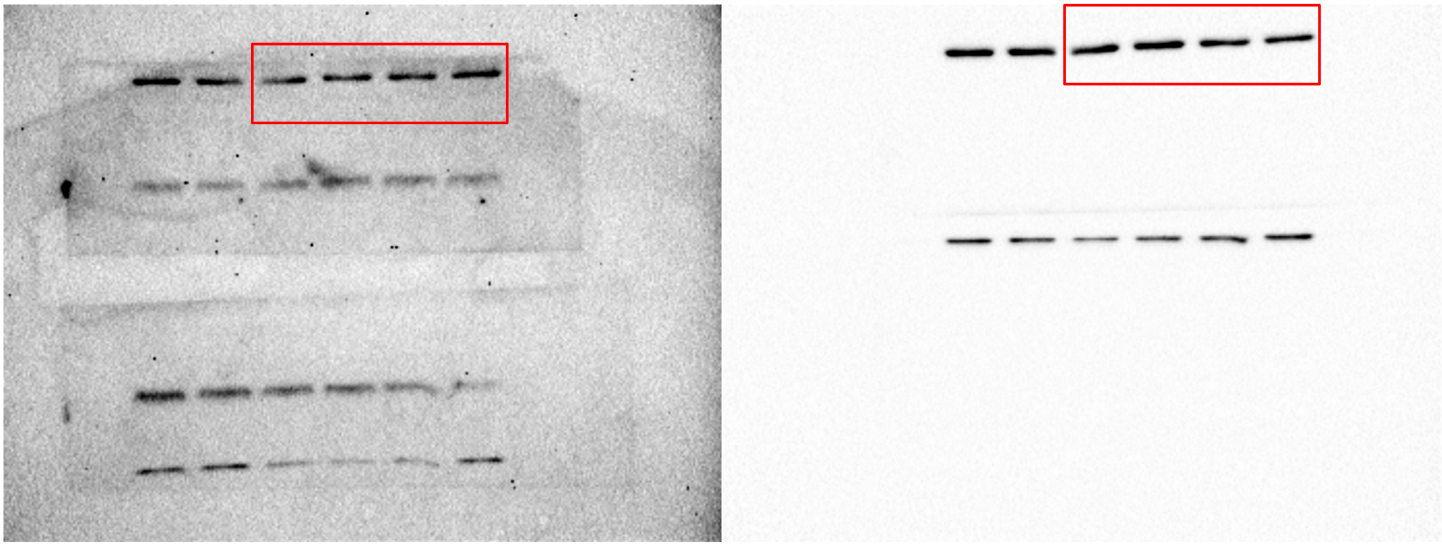

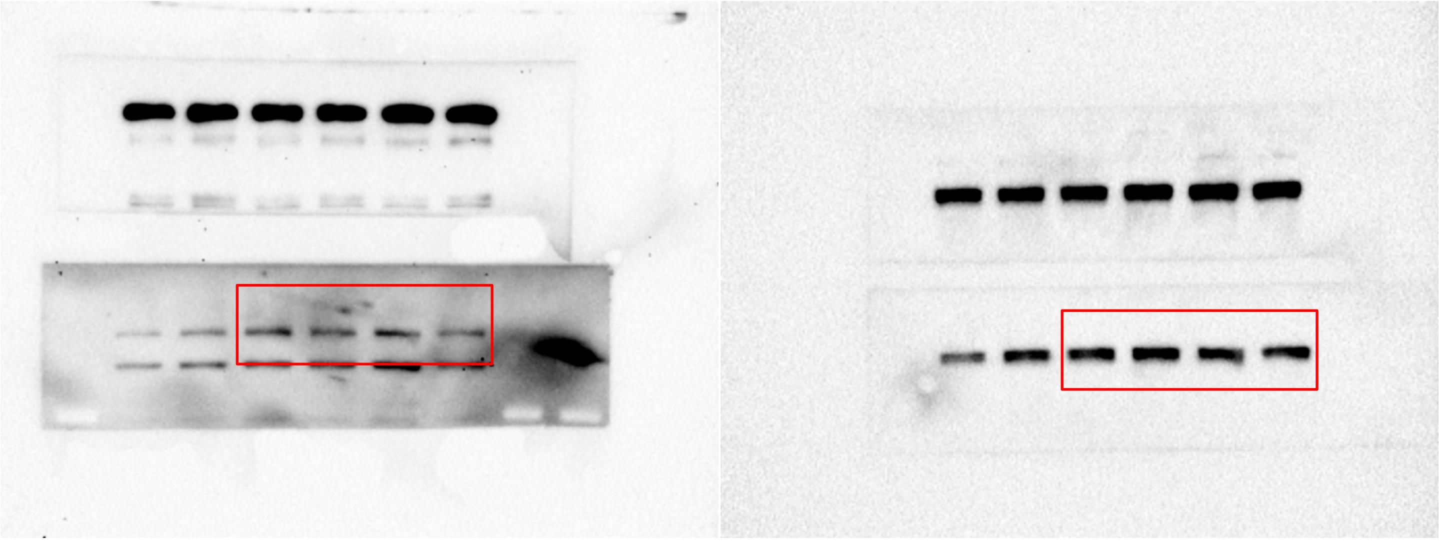

Unedited blot for Figure 4b

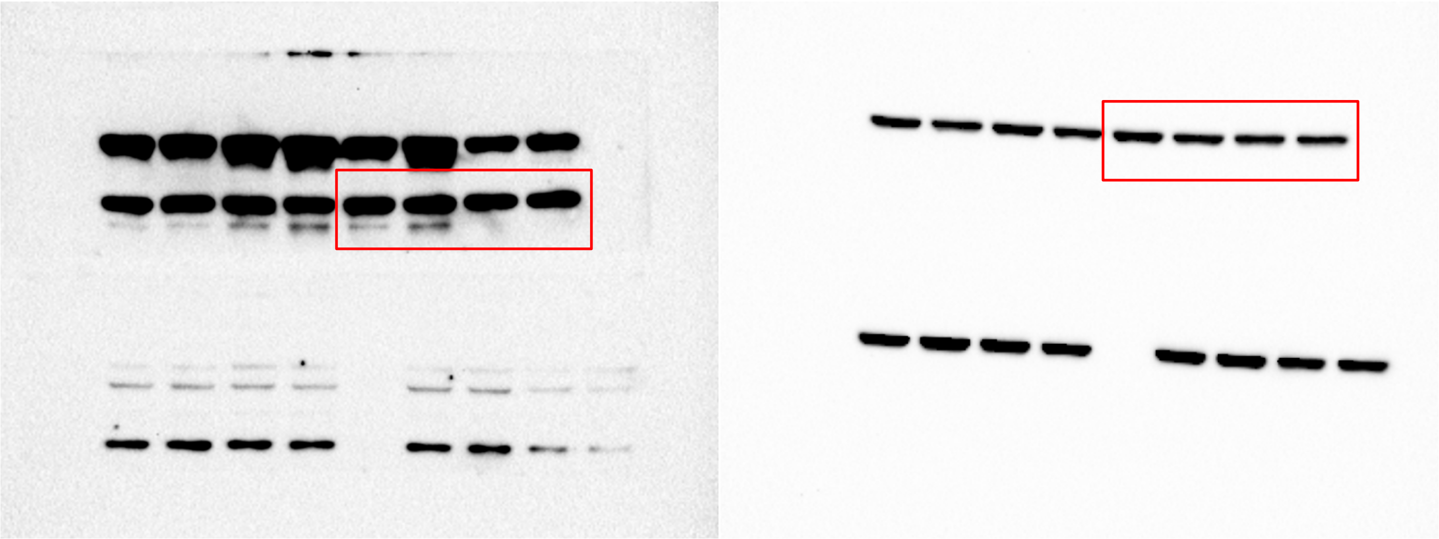

Unedited blot for Figure 4c

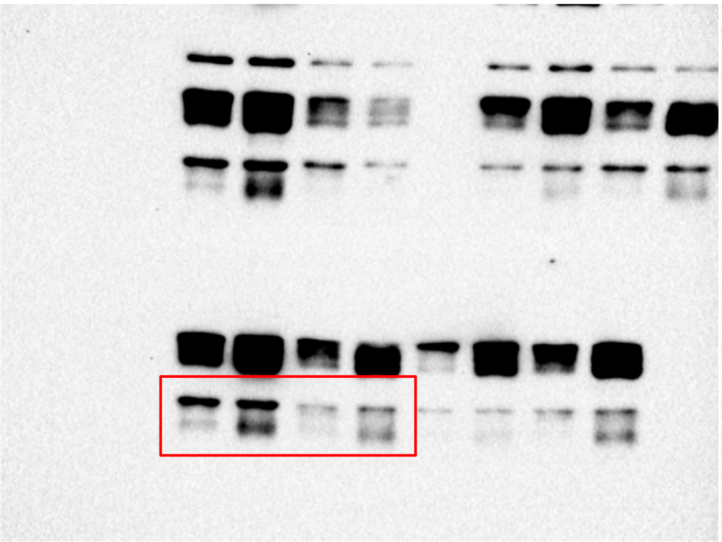

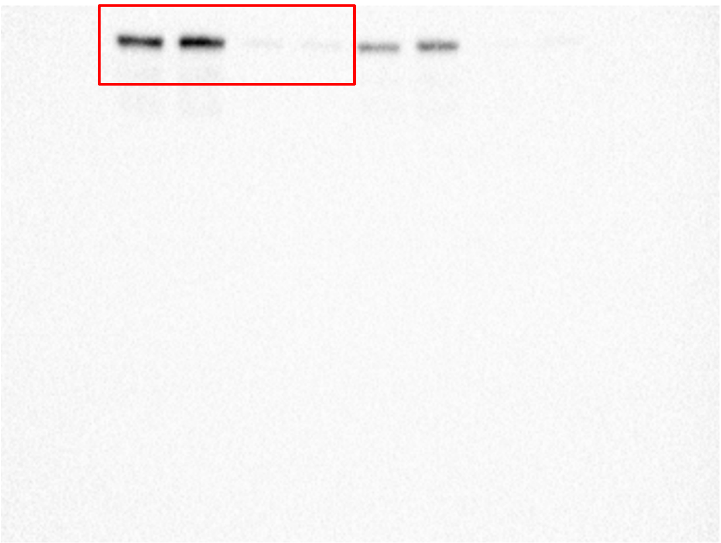

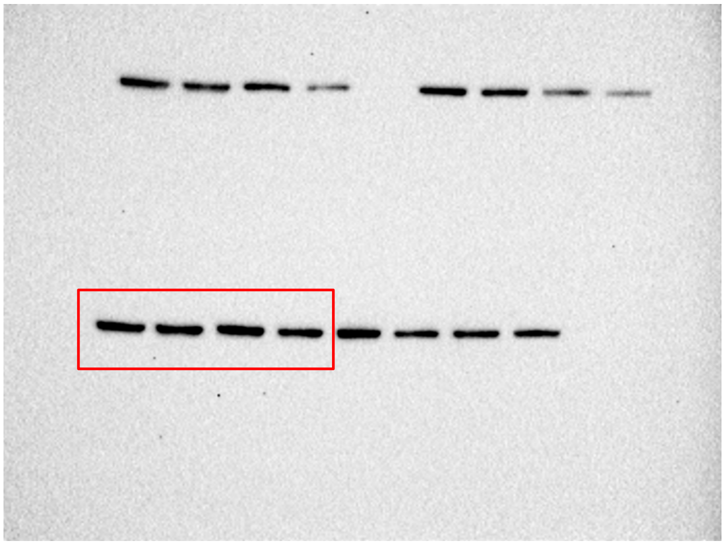

Unedited blot for Figure 4h

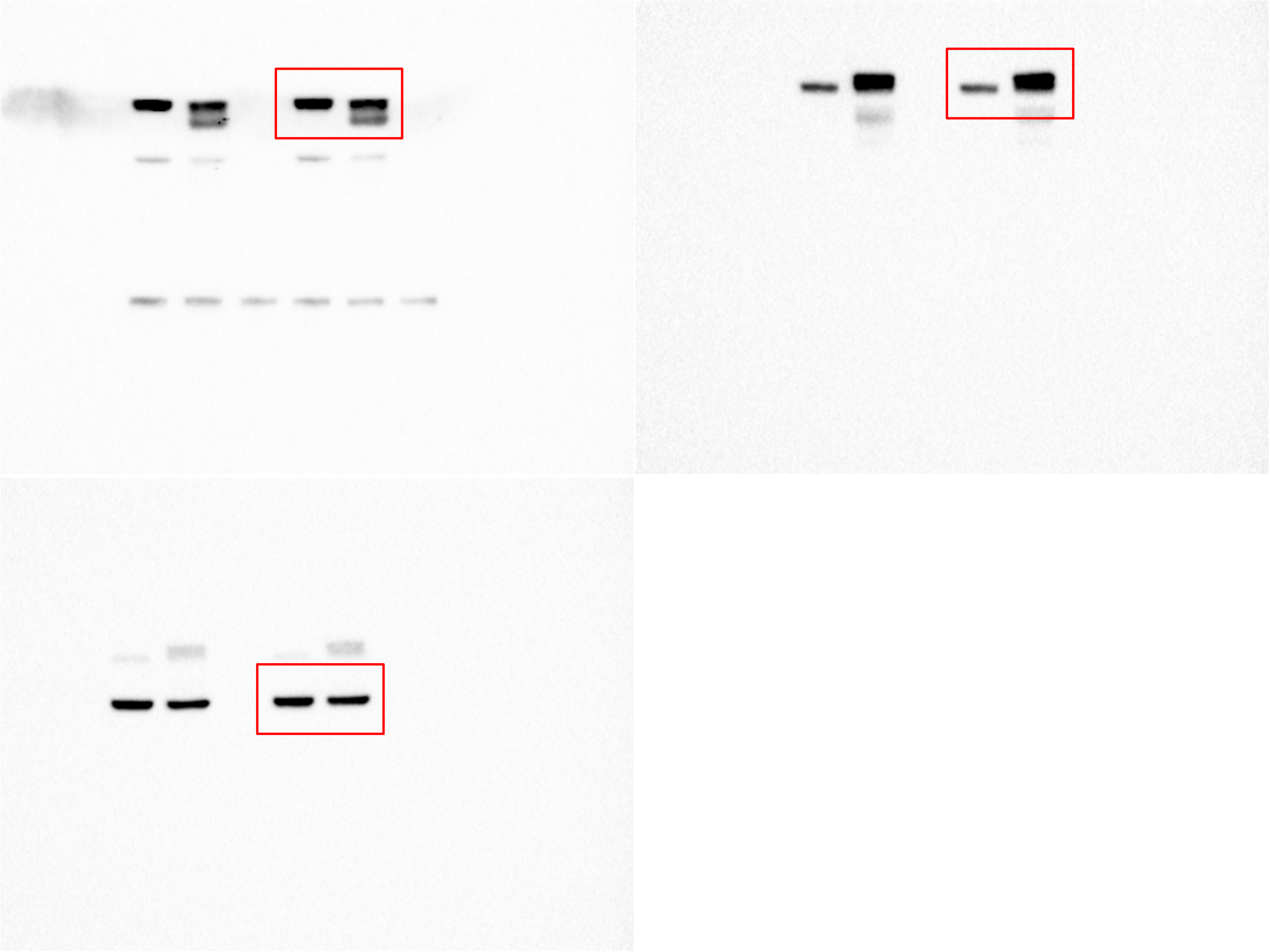

Supplementary Figure 9. Images for uncropped blots.
