# Supplementary material for: G protein-coupled estrogen receptor stimulates human trophoblast cell invasion via YAP-mediated ANGPTL4 expression
Source: Commun Biol. 2021 Nov 12;4:1285. doi: 10.1038/s42003-021-02816-5 (PMC8589964; doi:10.1038/s42003-021-02816-5)
Supplement: Supplementary file 4 — Reporting Summary [file 42003_2021_2816_MOESM4_ESM.pdf]

nature research

Corresponding author(s): Jung-Chien Cheng

Last updated by author(s): Oct 4, 2021

Reporting Summary

Nature Research wishes to improve the reproducibility of the work that we publish. This form provides structure for consistency and transparency in reporting. For further information on Nature Research policies, see our Editorial Policies and the Editorial Policy Checklist.

Statistics

For all statistical analyses, confirm that the following items are present in the figure legend, table legend, main text, or Methods section.

n/a	Confirmed	
[ ]	[x]	The exact sample size (n) for each experimental group/condition, given as a discrete number and unit of measurement
[ ]	[x]	A statement on whether measurements were taken from distinct samples or whether the same sample was measured repeatedly
[ ]	[x]	The statistical test(s) used AND whether they are one- or two-sided<br>*Only common tests should be described solely by name; describe more complex techniques in the Methods section.*
[x]	[ ]	A description of all covariates tested
[x]	[ ]	A description of any assumptions or corrections, such as tests of normality and adjustment for multiple comparisons
[ ]	[x]	A full description of the statistical parameters including central tendency (e.g. means) or other basic estimates (e.g. regression coefficient) AND variation (e.g. standard deviation) or associated estimates of uncertainty (e.g. confidence intervals)
[ ]	[x]	For null hypothesis testing, the test statistic (e.g. F, t, r) with confidence intervals, effect sizes, degrees of freedom and P value noted<br>*Give P values as exact values whenever suitable.*
[x]	[ ]	For Bayesian analysis, information on the choice of priors and Markov chain Monte Carlo settings
[x]	[ ]	For hierarchical and complex designs, identification of the appropriate level for tests and full reporting of outcomes
[x]	[ ]	Estimates of effect sizes (e.g. Cohen's d, Pearson's r), indicating how they were calculated

Our web collection on statistics for biologists contains articles on many of the points above.

Software and code

Policy information about availability of computer code

Data collection	No software was used for data collection.
Data analysis	R software, DAVID, Scion Image, Image-Pro Plus, and PRISM.

For manuscripts utilizing custom algorithms or software that are central to the research but not yet described in published literature, software must be made available to editors and reviewers. We strongly encourage code deposition in a community repository (e.g. GitHub). See the Nature Research guidelines for submitting code & software for further information.

Data

Policy information about availability of data

All manuscripts must include a data availability statement. This statement should provide the following information, where applicable:

- Accession codes, unique identifiers, or web links for publicly available datasets
- A list of figures that have associated raw data
- A description of any restrictions on data availability

All data generated or analyzed during this study are included in this manuscript and its supplementary information files.

Field-specific reporting

Life sciences study design

All studies must disclose on these points even when the disclosure is negative.

Sample size	In vitro experiments were performed at least for more than 3 times. Serum samples were collected from 17 PE patients and 16 normal pregnant women of similar age and gestational age. All statistical analyses were analyzed by PRISM software. For experiments involving only two groups, data were analyzed by t test. Multiple comparisons were analyzed using one-way ANOVA followed by Tukey's multiple comparison test. A significant difference was defined as p<0.05.
Data exclusions	No data were excluded.
Replication	In vitro experiments were performed at least for more than 3 times. All experiments were reproduced to reliably support conclusions stated in the manuscript.
Randomization	This study did not include animal or human participants. Thus, randomization was not relevant to our study.
Blinding	This study did not include animal or human participants. Thus, blinding was not relevant to our study .

Reporting for specific materials, systems and methods

We require information from authors about some types of materials, experimental systems and methods used in many studies. Here, indicate whether each material, system or method listed is relevant to your study. If you are not sure if a list item applies to your research, read the appropriate section before selecting a response.

Materials & experimental systems

n/a	Involved in the study	
☐	☒	Antibodies
☐	☒	Eukaryotic cell lines
☒	☐	Palaeontology and archaeology
☒	☐	Animals and other organisms
☒	☐	Human research participants
☒	☐	Clinical data
☒	☐	Dual use research of concern

Methods

n/a	Involved in the study	
☒	☐	ChIP-seq
☒	☐	Flow cytometry
☒	☐	MRI-based neuroimaging

Antibodies

Antibodies used	ANGPTL4 (WB, abcam: #ab206420, Clone name: EPR19873, Lot: GR276911-8), ANGPTL4 (IHC, Invitrogen: #40-9800, Clone name: N/A, Lot: UK294985), GPER (WB/IHC, abcam: #ab154069, Clone name: N/A, Lot: N/A), Phospho-YAP Ser127 (WB, Cell Signaling Technology: #13008, Clone name: D9W2I, Lot: 5), YAP (WB/IHC, Cell Signaling Technology: #12395, Clone name: D9W2I, Lot: 3), Phospho-LATS1 Ser909 (WB, Cell Signaling Technology: #9157, Clone name: D9W2I, Lot: 2), LATS1 (WB, Cell Signaling Technology: #3477, Clone name: C66B5, Lot: 7), Phospho-MST1 Thr183 (WB, Cell Signaling Technology: #49332, Clone name: E7U1D, Lot: 1), MST1 (WB, Cell Signaling Technology: #14946, Clone name: D8B9Q, Lot: 1), α-Tubulin (WB, Santa Cruz: #sc-23948, Clone name: B-5-1-2, Lot: C0520), Flag (IF, Sigma: #F1804, Clone name: M2, Lot: SLCD6338)
Validation	ANGPTL4 (abcam: #ab206420), GPER (abcam: #ab154069) and YAP (Cell Signaling Technology: #12395) were validated with specific siRNA by WB. All relevant results were presented in the manuscript.

Eukaryotic cell lines

Policy information about cell lines

Cell line source(s)	HTR-8/SVneo (ATCC, Lot: 70016636)
Authentication	HTR-8/SVneo cell line was authenticated by STR method.
Mycoplasma contamination	No mycoplasma contamination was detected.
Commonly misidentified lines (See ICLAC register)	HTR-8/SVneo was not listed in ICLAC database.
